# Molecular Design in l‑Glutamic Acid-Based Peptide Assembly Dynamics Driven by Carbodiimide-Fueled Reaction Cycle

**DOI:** 10.1021/acs.biomac.5c02255

**Published:** 2026-01-23

**Authors:** Nagihan Özbek, Xiaoyao Chen, Brigitte A. K. Kriebisch, Adrián Fernández-de-la-Pradilla, Katarzyna Świderek, Job Boekhoven, Beatriu Escuder

**Affiliations:** † Institute of Advanced Materials (INAM), 16748 Universitat Jaume I, 12071 Castelló, Spain; ‡ Department of Bioscience, School of Natural Sciences, 9184 Technical University of Munich, Lichtenbergstrasse 4, 85748 Garching, Germany

## Abstract

Molecular self-assembly creates complex structures through noncovalent interactions. Synthetic fuel-driven systems mimic biology, yet the effects of subtle design changes, particularly hydrophobic groups such as alkyl chains, are still not well understood. This study showed that the alkyl chain length critically influences the dynamic assembly of short peptides. Z-capped peptides **C3** and **C6**, composed of l-phenylalanine and -glutamic acid, with L-aspartic acid as the reactive site and alkylamide groups of varying lengths at the C-terminus, have been observed to form metastable aggregates via intramolecular anhydride formation during a chemically fueled reaction cycle. We elucidated that the difference in alkyl chain length resulted in either highly dynamic assemblies or delayed structural dissolution. Our findings provide a comprehensive understanding of these observations, illustrating how rational peptide design enable precise control over nanostructure properties and catalytic lifetimes.

## Graphical abstract

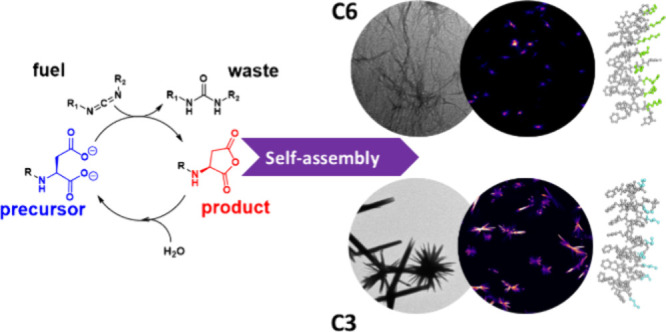

## 1 Introduction

Molecular self-assembly is a process in which supramolecular architectures are formed through noncovalent interactions, such as hydrogen bonds, van der Waals forces, and hydrophobic effects. This phenomenon has received considerable attention due to its potential as drug delivery vehicles,^1,2^ biomedical imaging technologies,^3^ and catalysis.^4^ Most synthetic supramolecular materials exist in or near an equilibrium state,^5–7^ which starkly contrasts biological assemblies. This fundamental difference underscores the distinctive nature and potential of synthetic self-assembled systems for diverse technological applications.

Short peptides are a promising building block for self-assembly as they are small, their assembly is easily tuned by the length of an alkyl chain, hydrophobic effects,^8^ intermolecular interactions,^9^ and the kinetics and thermodynamics of assembly.^10^ Their assembly is primarily driven by a balance between hydrophilic and hydrophobic interactions within the peptide molecules, with hydrophobic effects arising from nonpolar alkyl chains playing a pivotal role. Longer alkyl chains increase the surface area for molecular interactions, thereby enhancing van der Waals forces, π–π stacking, and hydrophobic effect, facilitating peptide assembly by promoting closer molecular alignment and packing, leading to more stable and ordered structures.^11–13^ Additionally, longer alkyl chains accelerate the assembly kinetics by providing more opportunities for intermolecular interactions and nucleation events, as enhanced hydrophobicity lowers the energy barrier for nucleation and promotes rapid growth of assembled structures.^14,15^ The self-assembly of short peptides arises from a delicate balance between enthalpic and entropic contributions. While extended alkyl chains enhance enthalpic van der Waals interactions, entropy-driven hydrophobic effects play a predominant role. This phenomenon promotes assembly stabilization by increasing overall entropy as water molecules are freed from their structured arrangements when hydrophobic areas aggregate. The interplay of these thermodynamic principles highlights the crucial importance of the hydrophobic effect in peptide self-assembly, with both enthalpic and entropic factors contributing to the process.^16–18^

Inspired by the nonequilibrium self-assembly often observed in biology, the dynamic nature of chemically fueled assemblies represents a rapidly evolving frontier in supramolecular chemistry. These assemblies exhibit unique material properties such as spatiotemporal control and self-healing capabilities owing to their dynamic activation and deactivation processes.^19,20^ In chemically driven self-assembly, a catalytic reaction cycle converts chemical fuel into waste while extracting energy. In an activation reaction, a precursor molecule reacts with the chemical fuel to form an activated product and a waste byproduct.^21,22^ This activated product can assemble into supramolecular structures. However, the activated product is inherently unstable and spontaneously deactivates, often through hydrolysis. Upon deactivation, the product reverts to the original precursor, completing the catalytic cycle. This cyclical process allows the precursor to catalyze fuel conversion repeatedly, generating transient building blocks for assembly when the precursor is soluble and the product assembles.

The dynamic activation and deactivation of the building blocks in these assemblies endows them with unique properties and new possibilities for applications that require controlled assembly and disassembly cycles. In biomedical applications, the ability to form and disassemble structures in response to specific stimuli can be harnessed for targeted drug delivery, where drug-carrying assemblies disassemble at the target site and precisely release therapeutic agents. In catalysis, the dynamic nature of these assemblies can enhance the efficiency and sustainability of catalytic processes by enabling repeated use of catalytic sites.^23^ Additionally, in the realm of smart materials, chemically fueled assemblies offer the potential to create responsive systems that can adapt to environmental changes, providing functionalities such as self-healing and adaptive responses. Overall, exploring molecular self-assembly, particularly the self-assembly of peptides and chemically fueled systems, offers profound insights and paves the way for designing advanced materials with tailored functionalities.^24^

One strategy to regulate self-assembly using a chemical reaction cycle is to temporarily neutralize molecular charges, thereby driving reversible self-assembly. In this process, activation converts a soluble anionic precursor into a neutral species, prompting assembly, while deactivation restores negative charges, triggering disassembly.^25^ We exemplified this process using EDC (1-ethyl-3-(3-(dimethylamino)­propyl) carbodiimide) to transform an anionic dicarboxylate into a neutral anhydride. The subsequent hydrolysis step regenerates the precursor, sustaining the cycle (Scheme 1).

**Scheme 1:**
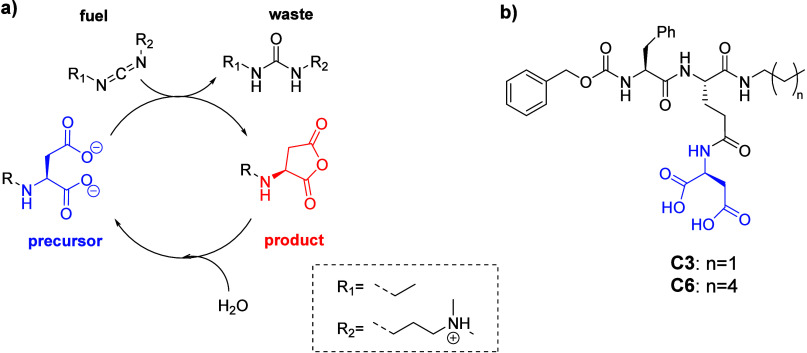
Schematic Representation of a Chemically-Driven Self-Assembly Process Regulated by Activation and Deactivation of Short Peptides (a) and Molecular Structure of the Precursors **C3** and **C6** (b)

Building on this approach, we aimed to create chemically fueled fibers by promoting intramolecular anhydride formation, transiently transforming carboxylate groups into a metastable anhydride. Here, we report two chemically fueled peptides, **C3** and **C6**, introducing an aspartic acid handle connected to glutamic acid γ-COOH as the fuel-responsive element. Both peptides are amidated at their C-termini with relatively short alkyl chains (N-propyl and N-hexyl). Previous chemically fueled peptide self-assembly systems predominantly employ a C-terminal aspartic acid as the reactive site for transient anhydride formation, whereby transient activation neutralizes the terminal charge and modulates assembly behavior.^26^ In contrast, our design places the reactive aspartic acid internally adjacent to an amidated glutamic acid, decoupling fuel-responsive chemistry from the peptide termini and shifting control of supramolecular packing toward sequence-defined noncovalent interactions. This original molecular design was inspired by our interest in understanding how variations in alkyl chain lengths, which modify hydrophobicity and van der Waals interactions as demonstrated in our previous work,^27^ along with the positioning of the reactive Asp group on a side chain that is responsible for the fiber rigidity,^28^ affect the nanostructures and lifetime of the catalytic cycle. Despite these minimal differences in molecular design, these peptides exhibit completely different reaction cycle kinetics, indicating a dramatic effect on self-assembly due to subtle structural differences. We demonstrate how a slight structural modification enables us to tune the lifetime of the catalytic cycle. We observed two distinct nanostructures when we fueled the precursors with 15 mM EDC. **C3** revealed a more rigid and collapsed form as rods than the longer alkyl chain length analog, **C6**. The peptides were characterized by nuclear magnetic resonance (NMR), Electrospray Ionization Mass Spectrometry (ESI-MS), and high-performance liquid chromatography (HPLC); the reaction cycles were followed by imaging techniques, including transmission electron microscopy (TEM) and confocal microscopy. To support the preliminary results, Molecular Dynamics simulations were performed to understand how the molecules are packing.

## 2 Results and Discussion

The peptides **C3** and **C6** were synthesized through solution-phase peptide synthesis and fully characterized. The peptide’s side chains p*K*_a_ were calculated to be 4.88 and 5.31, respectively, by potentiometric titration of 10 mM solutions (see SI). As we carry out our experiments in further work in an aqueous buffer of 200 mM MES at pH 6.0, the carboxylic acid groups are mostly deprotonated.

We started the reaction cycle by adding 15 mM EDC to the solution with the peptide at 2.5 mM, which converted the clear solution into a turbid hydrogel for both **C3** and **C6**. To understand the underlying chemical reaction kinetics of the system, we determined the concentrations of the activated and deactivated peptide. We used the benzylamine quench methodology,^29^ which freezes the cycle’s kinetics by converting the reactive anhydride into its stable amide and by raising the pH, which stops the activation. The concentration of benzylamide is thus used as a measure for anhydride concentration (activated peptide). We analyzed the kinetics of the chemical reaction cycle and measured its content by HPLC using EDC as the fuel and **C3**/**C6** as the precursors (Figure 1).

**Figure 1:**
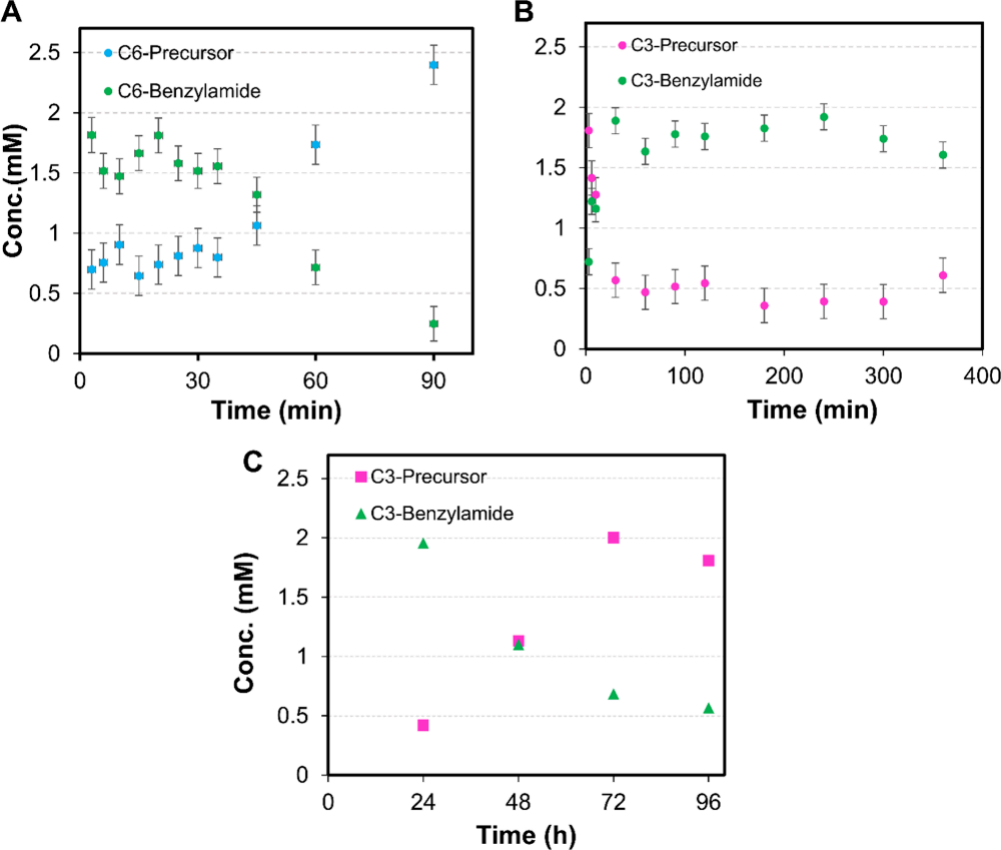
Analysis of the reaction kinetics by HPLC over time. Precursor and anhydride concentrations were calculated from the HPLC chromatograms at predetermined intervals for **C6** (A) and **C3** (B and C).

For this, following the reaction with EDC, aliquots of **C3** and **C6** were quenched at defined time intervals to monitor the progression of the reaction with EDC. To track the change in activated peptide concentration (benzylamide) over time, quenched samples were stored for 24 h and analyzed by HPLC. For **C6** (Figure 1A), the activated peptide was observed to form swiftly, reaching its maximum concentration at about 20 min, before diminishing and almost disappearing by the 90 min mark. This pattern suggests that the reaction is transient, implying the activated product loses stability once the fuel is consumed. Conversely, **C3** (Figure 1B) exhibited a more gradual and prolonged reaction. The activated peptide accumulates slowly, reaches its maximum at approximately 3 h, and then remains constant for several hours. To gain insight into its behavior over extended periods, the **C3** system was observed every 24 h for up to 96 h (Figure 1C). During this time, the activated peptide gradually deactivated, reducing to about 0.5 mM.

The process of assembly and disassembly was examined by monitoring the turbidity of the solution in response to EDC using a plate reader (Figure 2A,B), and the assembly’s composition was determined over time using ^1^H NMR spectroscopy (Figure 2C,D). While the hydrogel of the **C6**-based peptide converted to a transparent solution over 2 h, the **C3** gel dissolved much more slowly. Gradually, after 24 h, the turbidity decayed. To determine if the precursor was kinetically trapped, turbidity measurements were conducted every 24 h up to 120 h (See SI). Overall, a decrease in absorbance value was observed, which suggests that either the peptide disassembles very slowly after its deactivation (the assembly is metastable) or the deactivation reaction is very slow (the assembly protects the peptide from deactivation). The results obtained were supported by an assay with the solvatochromic dye Nile Red, indicating that the peptides were well-dissolved in their precursor state, as evidenced by the absence of a Nile Red signal (Figure S14).

**Figure 2:**
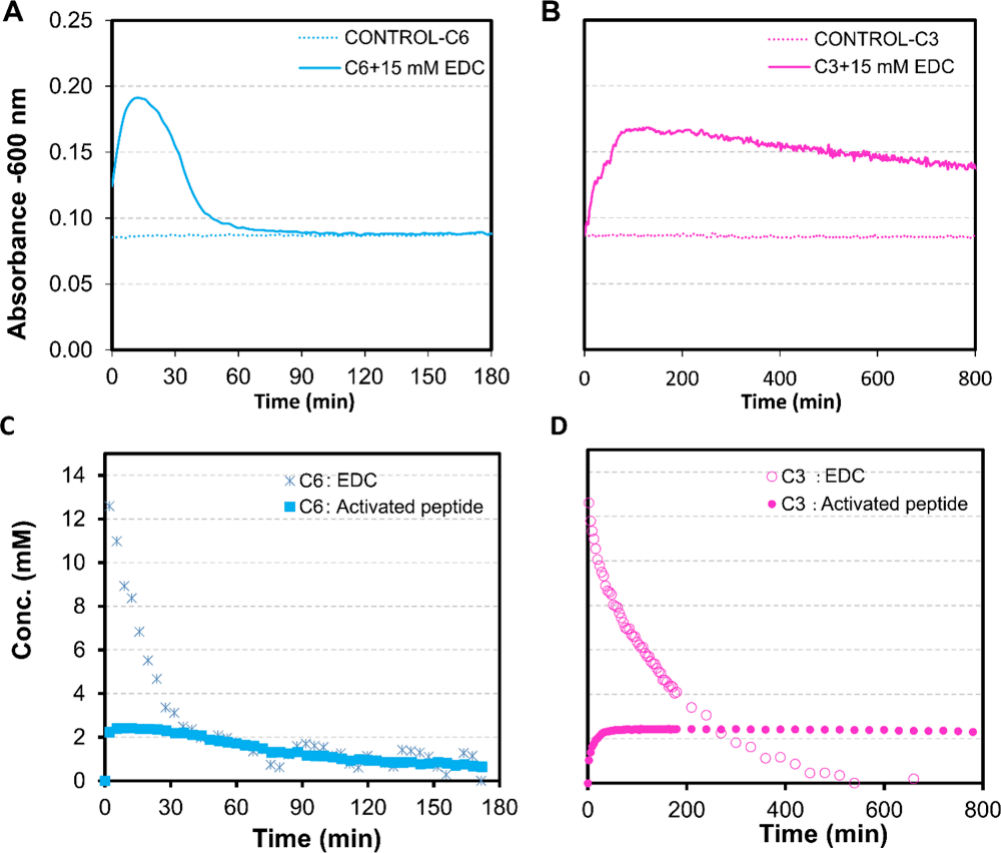
Turbidity assay of the 2.5 mM **C6** (A) and **C3** (B) solutions in 200 mM MES buffer at pH 6 in response to EDC at 600 nm and determination of the peptide assembly composition and EDC consumption using ^1^H NMR for **C6** (C) and **C3** (D) during the reaction cycle.

^1^H NMR spectroscopy study uses the fact that peptides within an assembly are NMR silent, thereby enabling the quantification of the initial 2.5 mM peptide present in the assemblies. By combining the ^1^H NMR spectroscopy and HPLC data, the ratio of precursor and activated product in the assemblies can be determined, assuming that all of the activated peptide is in the assembled state. However, 3 h after starting the reaction cycle, ^1^H NMR shows us the initial peptide concentration in the case of **C6** (Figure 2C), which points to nearly complete disassembly, whereas **C3** still remains assembled. ^1^H NMR results revealed that the initial peptide concentration for **C6** was consistent with complete disassembly after a 3-h reaction cycle, while **C3** (Figure 2D) still exhibited evidence of assembled structures. Additionally, selected ^1^H NMR spectra are presented to illustrate how the concentration of peptide incorporated into assemblies was estimated at representative time points during the reaction cycle (Figure S16).

The findings reveal a significant divergence in the behavior of the two systems: **C6** induces a rapid, temporary increase in product formation, whereas **C3** promotes slower, more sustained accumulation. The gradual decline in the activated product over several days suggests the slow hydrolysis of the activated peptide. This conclusion was corroborated by turbidity measurements, which also confirmed that the quenching method accurately captured these changes. Nonetheless, the reason why some precursors remain trapped and unreactive is not yet fully understood.

We hypothesized that due to the slow hydrolysis of **C3**, we would observe a more rigid, rod-like structure upon imaging (Figure 3). On the other hand, since anhydride hydrolysis is smooth for **C6**, this suggests a thinner fiber network. The TEM and confocal micrographs collectively illustrate the intricate nanofibrous morphology of **C3** and **C6** 10 min after fueling with EDC. For **C6**, the TEM image revealed a dense, fine network of elongated fibers, each less than 100 nm in diameter, indicating a highly interconnected structure. Whereas the TEM images of the **C3** display large, elongated and rigid fibers radiating from central points, extending several micrometers in length. Moreover, Nile Red dye has been applied to confocal microscopy, which is highly effective for visualizing hydrophobic regions. By using this dye, the distribution and formation of the fibers can be observed in detail, emphasizing the hydrophobic domains essential to the peptides self-assembly.

**Figure 3:**
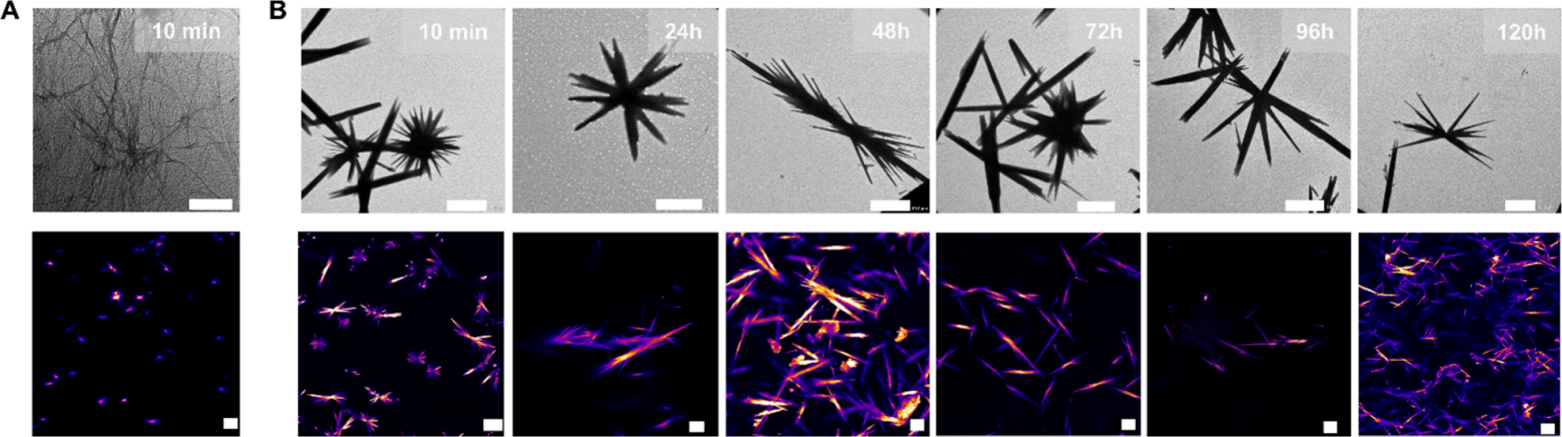
Morphological characterization of chemically driven peptide assemblies. Peptides **C3** and **C6** (2.5 mM each) were prepared in 200 mM MES buffer at pH 6 and fueled with 15 mM EDC. (A) **C6**: TEM (top) and confocal micrographs (bottom) acquired 10 min after EDC addition. Scale bars represent 1 μm. (B) **C3**: TEM (top) and confocal micrographs (bottom) acquired at 10 min and 24, 48, 72, 96, and 120 h after EDC fueling (images arranged from left to right). All scale bars represent 10 μm.

The confocal micrographs corroborate the presence of a rigid rod network for **C3** and a dense fiber network for **C6** (Figure 3), while TEM reveals the nanometer details of these nanostructure networks. Thus, our chemical reaction cycle converts solutions into hydrogels at the expense of chemical energy. Additionally, our analysis of the TEM and confocal micrographs of **C3** over a period of up to 120 h (Figure 3B) indicated notable morphological changes in the nanostructures of **C3** during this time frame.

Initially, at 24 h, the structures are predominantly star-shaped, displaying sharp and well-defined features, indicating early nucleation and growth. However, as time advances to 48 h, the nanostructures transform into rod-like or needle-like shapes, exhibiting directional growth. By 72 h, the structures become more complex and abundant, showcasing extensive branching and aggregation. At 96 h, the structures display significantly enhanced growth and a greater degree of aggregation, with evidence of nanostructure fusion. Finally, at 120 h, the structures form dense, interconnected networks, reflecting advanced stages of growth and self-assembly. Throughout this period, the key changes include an increase in the length and branching of the structures, greater complexity and density, and enhanced aggregation, which suggest a continuous process of nucleation, growth, and structural maturation. On the contrary, the TEM micrograph of **C6**, 30 min after the addition of EDC, shows elongated, fibrous nanostructures with diameters ranging roughly from 50 to 200 nm (Figure S17). These fibers are generally smooth but exhibit subtle signs of twisting, creating a network-like structure. The scanning electron microscopy (SEM) image in the middle confirms these observations, depicting a dense and intertwined network of fibers with consistent diameters. This image also emphasizes the twisted nature of the fibers, implying a potential growth mechanism that involves twisting and bundling.

Using Attenuated Total Reflectance Fourier-transform infrared spectroscopy (ATR–FTIR), we investigated the impact of varying alkyl chain lengths on the β-sheet interactions present in the fibers. In ATR–FTIR measurements of lyophilized samples, β-sheet interactions are typically characterized by amide I bands in the 1625–1640 cm^–1^ range (Figure 4; full spectra shown in Figure S18).

**Figure 4:**
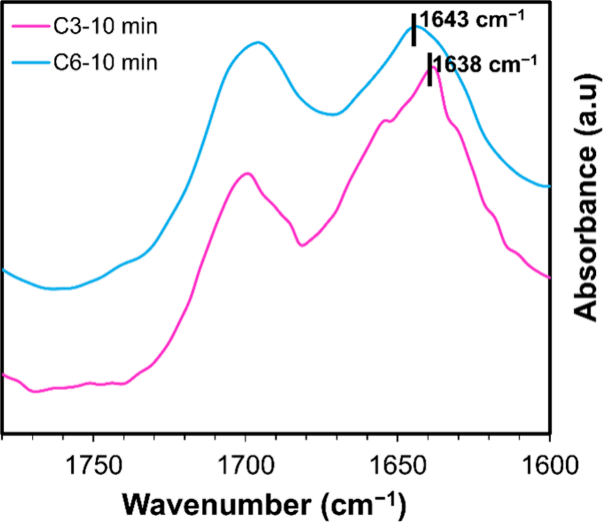
Selected region of ATR-FTIR spectra of **C3** and **C6** at 10 min after fueling with EDC.

These fibers showed two distinct peaks for **C3** and **C6**: the amide I band at 1638 cm^–1^ for **C3** and 1643 cm^–1^ for **C6**. Furthermore, the change in the amide I band position from 1638 cm^–1^ - **C3**, to 1643 cm^–1^ - **C6** sequence, in infrared spectroscopy is indicative of the sensitivity of this band to hydrogen bonding within peptide arrangements. As the peptide assemblies become less ordered, particularly when the β-sheet interactions between the peptides are reduced, the amide I band broadens and shifts toward higher wavenumbers. These findings complement our imaging results, as **C6** creates less ordered and more dynamic nanostructures, fibers, while **C3** represents rods with ordered and rigid arrangements.^28,30^ The band around 1697 cm^–1^ is assigned to both peptides’ C=O stretching vibrations of the Z-OCNH (carbamate) group.

We used molecular dynamics (MD) simulations to elucidate further the differences in the assembly behavior of these two peptides.^31–33^ For our monomers, parallel configurations enable them to establish more hydrogen bond interactions and maintain them for longer periods than antiparallel arrangements. Our experimental data from infrared spectroscopy on **C3** and **C6** monomers support this conclusion and suggest the presence of parallel β-sheets. To investigate the stability of parallel and antiparallel β-sheet arrangements, we designed a model using the depicted representation (Figure 5).

**Figure 5:**
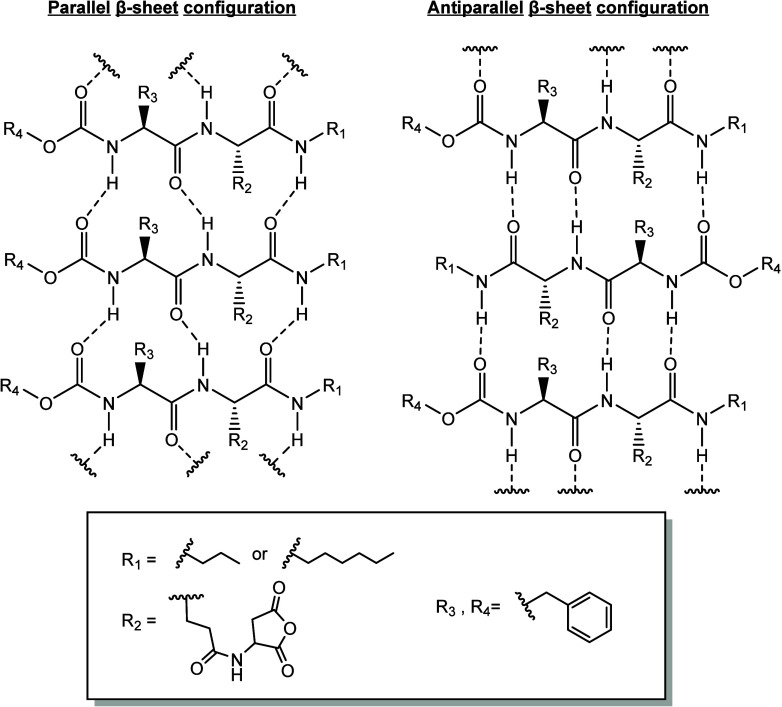
Chemical structure of possible parallel and antiparallel β-sheet configurations for **C3** and **C6**.

By studying the evolution of the four possible configurations of the **C3** and **C6** monomers during 40 ns of molecular mechanics/molecular dynamics (MM/MD) simulations, we gained a deeper understanding of which one could be the origin of the experimentally observed parallel arrangement preference. The time evolution of the root-mean-square deviations (RMSDs) for the central β-sheet during the unconstrained simulations (See SI) shows that from 30 ns on, the systems are mainly equilibrated. Therefore, the 30–40 ns interval was chosen for analyzing the preservation of hydrogen bond contacts (Figure 6). As can be observed from the plots, both parallel β-sheets exhibited a higher number of hydrogen bonds throughout the MD simulations, resulting in a more stable secondary structure of the aggregate. It is also true that some contacts are more preserved in antiparallel configurations. However, when all the frames in which hydrogen bonds appear are added, we get 5948 for **C3**-parallel, 5151 for **C3**-antiparallel, 10,260 for **C6**-parallel, and 7732 for **C6**-antiparallel. Thus, parallel configurations allow monomers to establish a higher number of hydrogen bonds, as well as maintain them for a longer period than antiparallel arrangements. This conclusion is in agreement with our experimental data from infrared spectroscopy, suggesting that **C3** and **C6** monomers form parallel β-sheets. Another relevant information extracted from this analysis is that among the most preserved H-bonds are those between the carbonyl of the phenylalanine (Phe) residue and the nitrogen of the backbone of the modified glutamic acid residue (RNG). In **C3**-parallel, we observed six of these interactions, and in **C6**-parallel, eight interactions appeared, all of which occurred up to 30% of the time. Other relevant contacts are between the N–H group of Phe and the carbonyl of the carboxybenzyl (CBZ) subunit, as well as some between the amide with the **C3** or **C6** chain (CHN or CHM) and the carbonyl of RNG.

**Figure 6:**
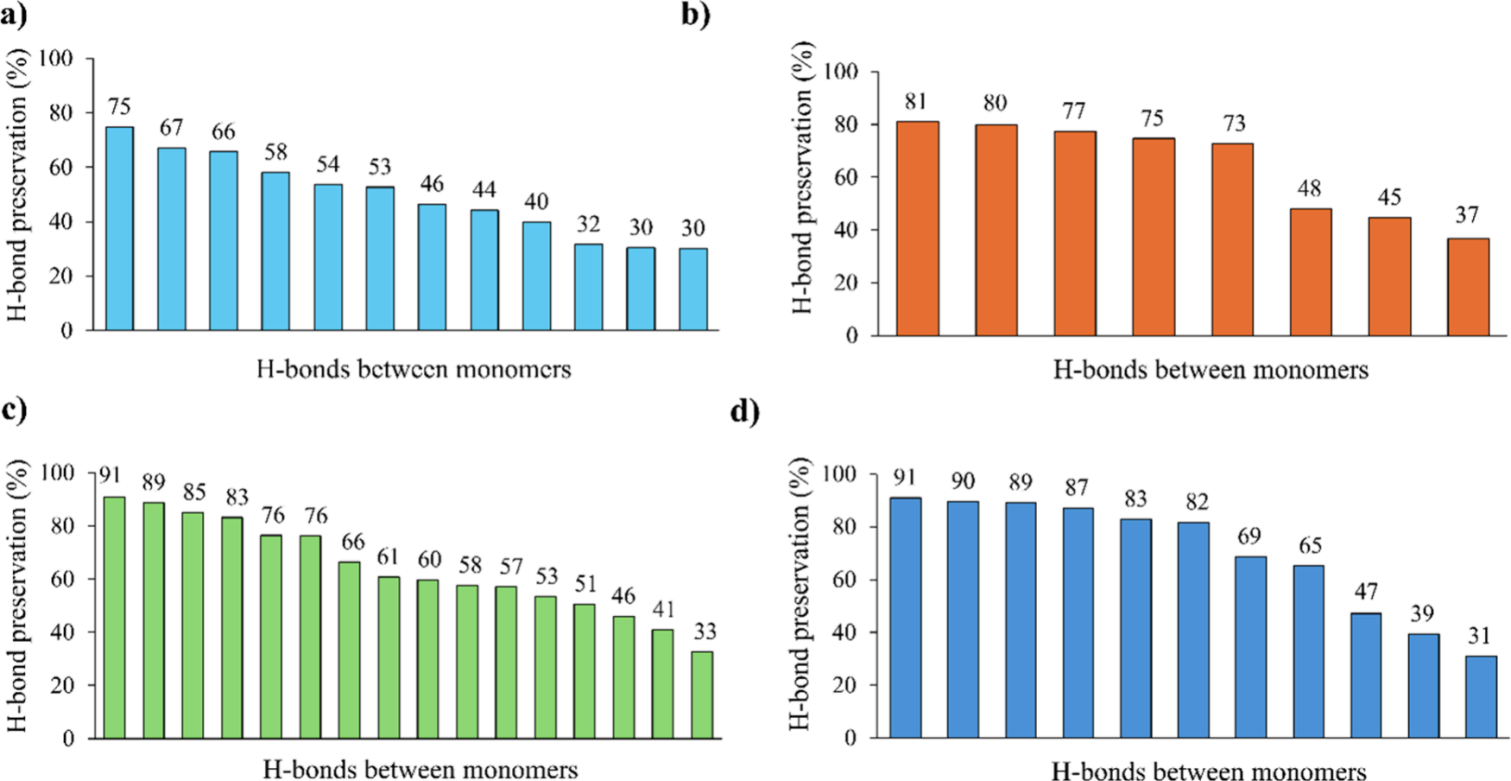
H-bonds between monomers of the central β-sheet with (a) **C3**-parallel, (b) **C3**-antiparallel, (c) **C6**-parallel, and (d) **C6**-antiparallel configurations based on 1000 snapshots generated during the last 10 ns of the MD simulation. Each bar represents one hydrogen bond contact between two monomers, and the label on top shows the percentage of time during the MD simulations that this H-bond is formed. Data was obtained defining 3.0 Å as the cutoff distance between the hydrogen donor and acceptor and 135° as the angle.^34^

Lastly, observing the disposition of the alkyl chains in a parallel and in an antiparallel configuration (Figure 7) could shed light on the reason why the **C3** and **C6** monomers aggregate differently despite having the same type of β-sheet arrangement. When the arrangement is parallel (Figure 7a,c), the alkyl chains are all oriented toward the same side, while if it is antiparallel (Figure 7b,d), each chain is wrapped in between the benzyl rings of the neighboring CBZ groups. Therefore, the alkyl chains have more freedom of movement in a parallel configuration, as well as if the chain is longer. For this reason, a longer alkyl chain, as in C6 monomer, will expectably introduce more entropy to the aggregation process, which will lead to less packed aggregates than those originating from **C3** monomer. Consequently, together with the exposition and mobility of the alkyl chains, the aggregate would become less packed as the alkyl chain gets bigger. Hence, the β-sheet with a smaller aliphatic chain (**C3**) would show a more packed, crystal-like structure than **C6**, which is in agreement with the observations coming from CM.

**Figure 7:**
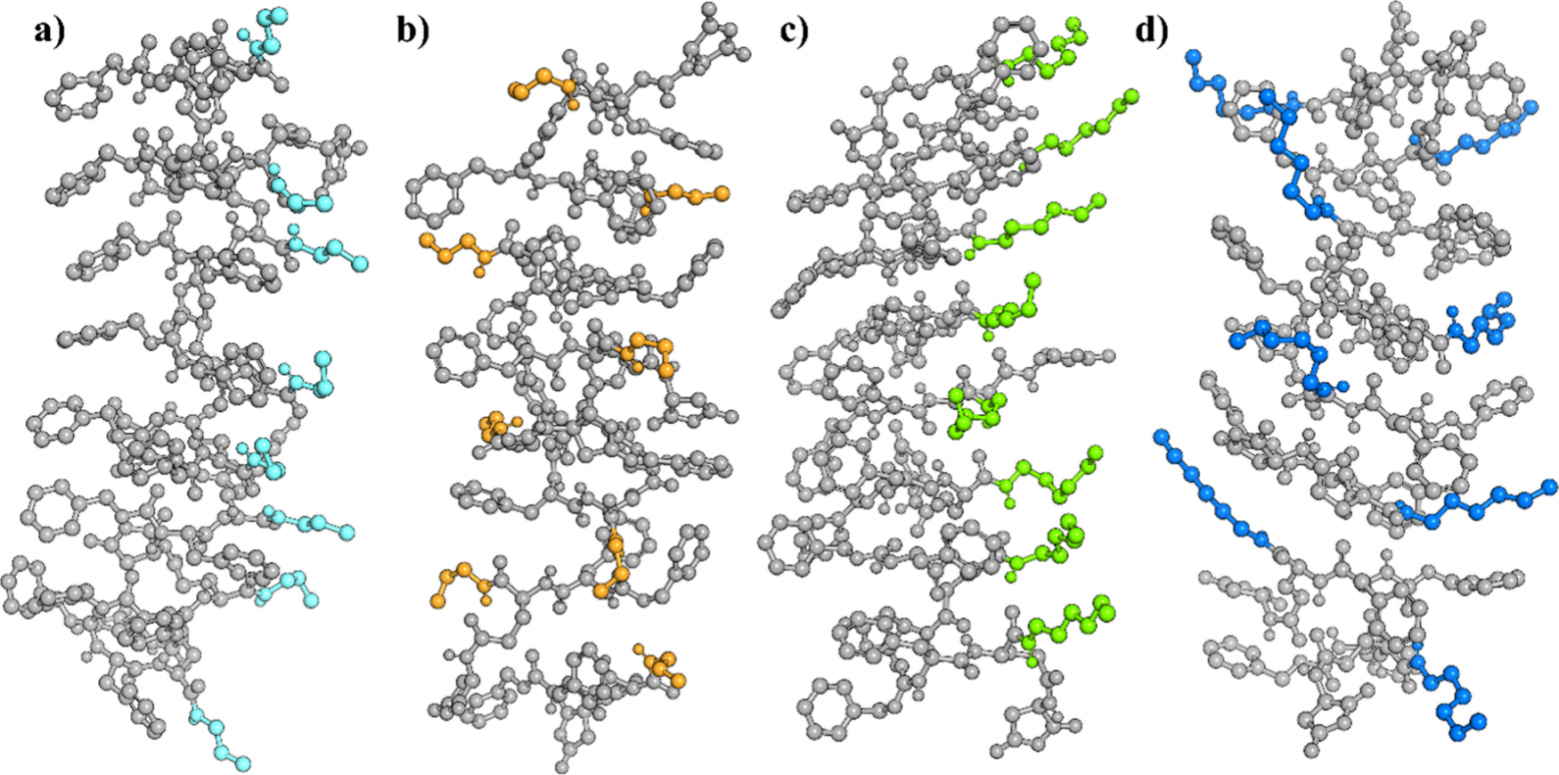
Snapshot of the last frame of the unconstrained MD simulations showing the disposition of the monomers of the central β-sheet in each configuration with the aliphatic chains highlighted: **C3** (a) and **C6**-parallel (c) alkyl chains in light blue and green and **C3** (b) and **C6**-antiparallel (d) alkyl chains in orange and marine blue, respectively.

In order to explain **C3** and **C6** macroscopical behavior, a more complex simulation was performed. The goal of this MD was to tackle two key questions: whether the length of the alkyl chains or the orientation of the β-sheets affects the stability of the interphase. With this goal in mind, we built for each monomer a system of three β-strands with the central one in two possible orientations, parallel to the others or flipped 180° (Figure S20). To guarantee stability of each strand during the simulations without losing mobility at the interphases, we constrained the most preserved H-bond in these β-sheets, between the backbone of the anhydride residue and the carbonyl of the phenylalanine, located at the center of each monomer. The MD simulations lasted for 10 ns, and after plotting the RMSD (Figure S21) we observed that the system is equilibrated after 5 ns, so this is the interval chosen for the interphase analysis.

The nonbonding interactions between each end of the 8 residues of the central chain and the ends of each side were computed and averaged, as shown in Figure 8.

**Figure 8:**
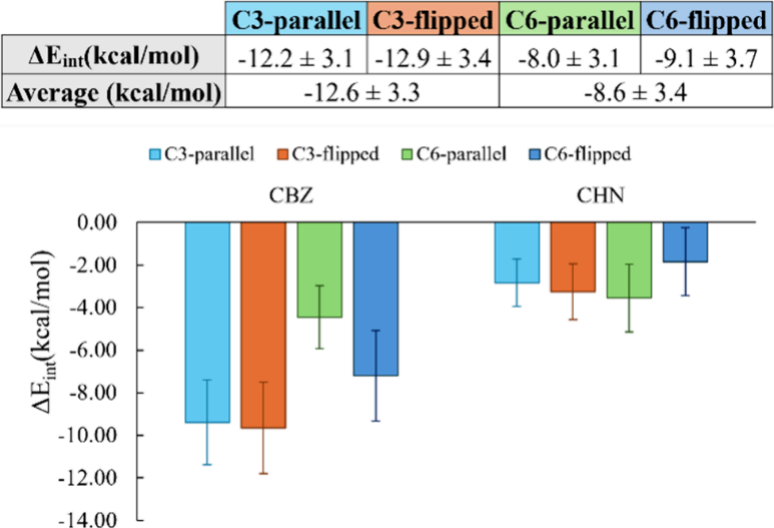
Average interaction energy (*E*_elec_ + *E*_LJ_) given in kcal/mol for both ends individually, and the total of the central monomers for each β-sheet arrangement, based on 1000 snapshots generated during the last 5 ns of the MD simulation. The uncertainty associated with the computed values is shown as error bars.

The results show no preference for a parallel or flipped conformation in terms of interactions at the interphase. This result is clearer for **C3**, in which the interactions of both ends are equivalent independently of the configuration, while in **C6**-flipped, the interactions of the CBZ moiety are higher and the CHN lower with respect to the **C6**-parallel configuration. However, when summing these interactions, the total is almost equivalent (−12.2 kcal/mol vs −12.9 kcal/mol and −8.0 kcal/mol vs −9.1 kcal/mol), so we cannot determine if the arrangement of the interphases of β-sheets is in one or the other direction.

Nonetheless, **C3** interphase is significantly more stable than the **C6** interphase, regardless of the chosen configuration. To prove that, the energy values of both configurations for each monomer were averaged, giving a value of −12.6 kcal/mol for **C3** and −8.6 kcal/mol for **C6**. This difference of 4 kcal/mol in just one interphase indicates that in an aggregate composed of thousands of these β -sheets, the gap between **C3** and **C6** interphases is going to be much bigger. Given this, one could hypothesize that **C3** would form a highly packed aggregate with low exposition to the solvent, while the **C6** aggregate would have a weaker interphase, much more accessible to the solvent. This is exactly what was observed under confocal microscopy and demonstrated in the kinetic studies: the **C3** aggregate is significantly more stable and forms rods, whereas **C6** is easily hydrolyzed and forms fibers.

## 3 Conclusions

We investigated how molecular design influences feedback and aggregation in chemically driven peptide assemblies, focusing on fibers and rods. By tuning the alkyl chain length, we controlled the rigidity and internal organization of two peptide analogs, modulating their preference for parallel or antiparallel β-sheet hydrogen bonding. Structural trends mirror those in **C3** and **C6** assemblies; longer hydrophobic chains yield more flexible, solvent-accessible structures that accelerate hydrolysis, while shorter chains promote rigidity. Similar behavior has been reported for other amphiphilic peptides and phosphate ester–fueled assemblies, where solvent exposure and fuel structure influence supramolecular organization.^35^ Our findings suggest that alkyl chain hydrophobicity and spatial constraints shape assembly lifetimes and morphologies. We are probing whether these differences arise from electrostatics or secondary structure. These insights offer design principles for dynamic, chemically fueled assemblies from short peptides.

## Supplementary Material



## Notes

Additional details on the materials and methods, including NMR, mass spectrometry, ATR-FTIR, turbidity measurements, and computational methods (PDF)

## Abbreviations

NMR: nuclear magnetic resonance
HPLC: high performance liquid chromatography
TEM: transmission electron microscopy
SEM: scanning electron microscopy
ATR-FTIR: attenuated total reflectance Fourier-transform infrared spectroscopy
