# Supplementary material for: Molecular Design in l‑Glutamic Acid-Based Peptide Assembly Dynamics Driven by Carbodiimide-Fueled Reaction Cycle
Source: Biomacromolecules. 2026 Jan 23;27(2):1690–7. doi: 10.1021/acs.biomac.5c02255 (PMC12892325; doi:10.1021/acs.biomac.5c02255)
Supplement: Supplementary file 1 [file bm5c02255_si_001.pdf]

Supporting Information (SI) for

Molecular Design in L-Glutamic Acid-Based Peptide Assembly Dynamics Driven by Carbodiimide-Fueled Reaction Cycle

Nagihan Özbek,[a] Xiaoyao Chen,[b] Brigitte A. K. Kriebisch,[b] Adrián Fernández-de-la-Pradilla,[a] Katarzyna Świderek,[a] Job Boekhoven*[b] and Beatriu Escuder*[a]

a Institute of Advanced Materials (INAM), Universitat Jaume I, 12071 Castelló, Spain.

Email: escuder@uji.es

b Department of Bioscience, School of Natural Sciences, Technical University of Munich, Lichtenbergstrasse 4, 85748 Garching, Germany.

Email: job.boekhoven@tum.de

Materials and Methods

1. Peptide synthesis and characterizations

Synthesis of $ZFEC_3$ / $ZFEC_6$. The dipeptide derivatives $ZFEC_3$ and $ZFEC_6$ used in this study, were synthesized using solution phase peptide synthesis as described in **Scheme S1.**[1]

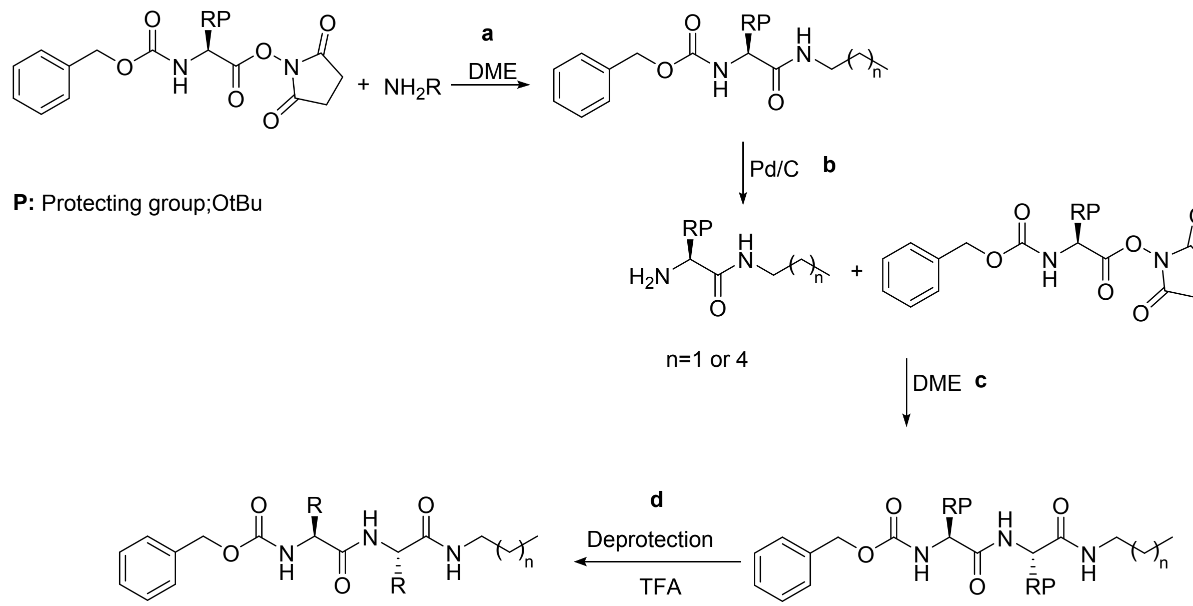

Scheme S1. Overview of the dipeptide synthesis **a**) coupling reaction between an activated carboxyl group and a free amine group, **b**) N-benzyloxycarbonyl deprotection, **c**) Coupling of the second amino acid, **d**) Deprotection of the t-butyl ester (OtBu).

a) Coupling reaction: The N-hydroxysuccinimide ester (1 eq.) was dissolved in DME, then the corresponding amine (1.2 eq.) was dissolved in DME and added dropwise. The resulting solution was stirred overnight. The solvent was evaporated by rotary, and the resulting solid (white matter or oil) was dissolved in CH_2Cl_2 and washed with 0.1 M Na_2CO_3, 0.1 M HCl and water respectively. The final product was dried under vacuum.

b) N-benzyloxycarbonyl deprotection: The corresponding N-benzyloxycarbonyl protected peptide derivative and catalytic amount of Pd over activated carbon (5-10 % w/w) were placed in a two-necked round bottom flask and suspended in MeOH. The system was purged to remove air with N_2 and connected to an H_2 atmosphere, and the black-grey suspension was continued for 24h in total. The black suspension was filtered over Celite and the solvent was evaporated by rotary, the resulting oil/matter was dried further under vacuum.

c) Coupling of the second amino acid: The N-hydroxysuccinimide ester (1.1 eq.) was dissolved in DME, and the corresponding amine with the C-terminal group (1 eq.) was also dissolved in DME. The mixture was stirred at r.t. overnight. If a concentrated precipitate formed, it was filtered using a

crucible and sequentially washed with 0.1 M Na_2CO_3, 0.1 M HCl, and water. For oily matter, it was dissolved in CH_2Cl_2 and washed similarly. The final product was vacuum-dried.

d) Deprotection: The t-butyl ester (OtBu) protected dipeptide was dissolved in a TFA-dichloromethane-water mixture and stirred for 40 minutes at r.t. After evaporating TFA and dichloromethane under reduced pressure, the residue was co-distilled with Et_2O, washed with water, and dried under vacuum.

ZFEC$_3$: ^{1}H NMR (400 MHz, 30 °C, DMSO-d_6) δ (ppm) = 12.16 (s, 1H, -COOH; H_h), 8.04 (d, J = 8.0 Hz, 1H, N-H; H_m), 7.78 (t, J = 5.6 Hz, 1H, N-H; H_d), 7.49 (d, J = 8.4 Hz, 1H, N-H; H_i), 7.35 – 7.15 (m, 10H, Ph-H; H_o and H_l), 4.96 (s, 2H, Ph-CH_2-O; H_n), 4.39 – 4.17 (m, 2H, H_j and H_e), 3.09 – 2.92 (m, 3H, H_k and H_c), 2.82 – 2.66 (m, 1H, $H_{k'}$), 2.22 (m, 2H, H_g), 1.97 -1.69 (m, 2H, H_f), 1.45-1.33 (m, 2H, H_b), 0.83 (t, J = 7.4 Hz, 3H, H_a). ^{13}C NMR (101 MHz, 30 °C, DMSO-d_6) δ 175.1 (-COO; C_{11}), 173.6 (C_{12}), 171.7 (C_7), 156.9 (C_3), 137.0 (C_1), 136.7 (C_6), 129.0 -126.4 (Ph-10C; C_{16-25}), 66.1 (C_2), 56.6 (C_4), 52.7 (C_8), 40.9 (C_{13}), 37.4 (C_5), 29.7 (C_{10}), 27.1 (C_9), 22.1 (C_{14}), 10.3 (C_{15}). ESI-TOF, positive mode: m/z $C_{25}H_{31}N_3O_6$ $[M+H]^+$ 470.22 $[M+H]^+$, 492.21 $[M+Na]^+$, 508.18 $[M+K]^+$.

ZFEC$_6$: ^{1}H NMR (400 MHz, 30 °C, DMSO-d_6) δ (ppm) = 12.11 (s, 1H, -COOH; H_k), 8.03 (d, J = 8.0 Hz, 1H, N-H; H_l), 7.77 (t, J = 5.6 Hz, 1H, N-H; H_g), 7.50 (d, J = 8.5 Hz, 1H, N-H; H_p), 7.36 – 7.17 (m, 10H, Ph-H; H_r and H_o), 4.97 (s, 2H, Ph-CH_2-O; H_q), 4.36 – 4.19 (m, 2H, H_m and H_h), 3.15 – 2.95 (m, 3H, H_n and H_f), 2.81 – 2.70 (m, 1H, $H_{n'}$), 2.21 (m, 2H, $H_{i'}$ and H_j), 1.96 -1.85 (m, 1H, $H_{j'}$), 1.82 -1.70 (m, 1H, H_i), 1.43-1.34 (m, 2H, H_e), 1.32-1.21 (m, 6H, H_{b-d}), 0.85 (t, J = 7.4 Hz, 3H, H_a). ^{13}C NMR (101 MHz, 30 °C, DMSO-d_6) δ 174.3 (-COO; C_{11}), 171.9 (C_{12}), 171.1 (C_7), 156.3 (C_3), 138.5 (C_1), 137.5 (C_6), 129.7 -126.5 (Ph-10C; C_{19-28}), 65.8 (C_2), 56.7 (C_4), 52.5 (C_8), 38.9 (C_{13}), 37.9 (C_5), 31.5 (C_{10}), 30.6 (C_9), 29.4 (C_{14}), 28.1 (C_{15}), 26.5 (C_{16}), 22.5 (C_{17}), 14.3 (C_{18}). ESI-TOF, positive mode: m/z $C_{28}H_{37}N_3O_6$ $[M+H]^+$ 512.28 $[M+H]^+$, 534.25 $[M+Na]^+$, 550.23 $[M+K]^+$.

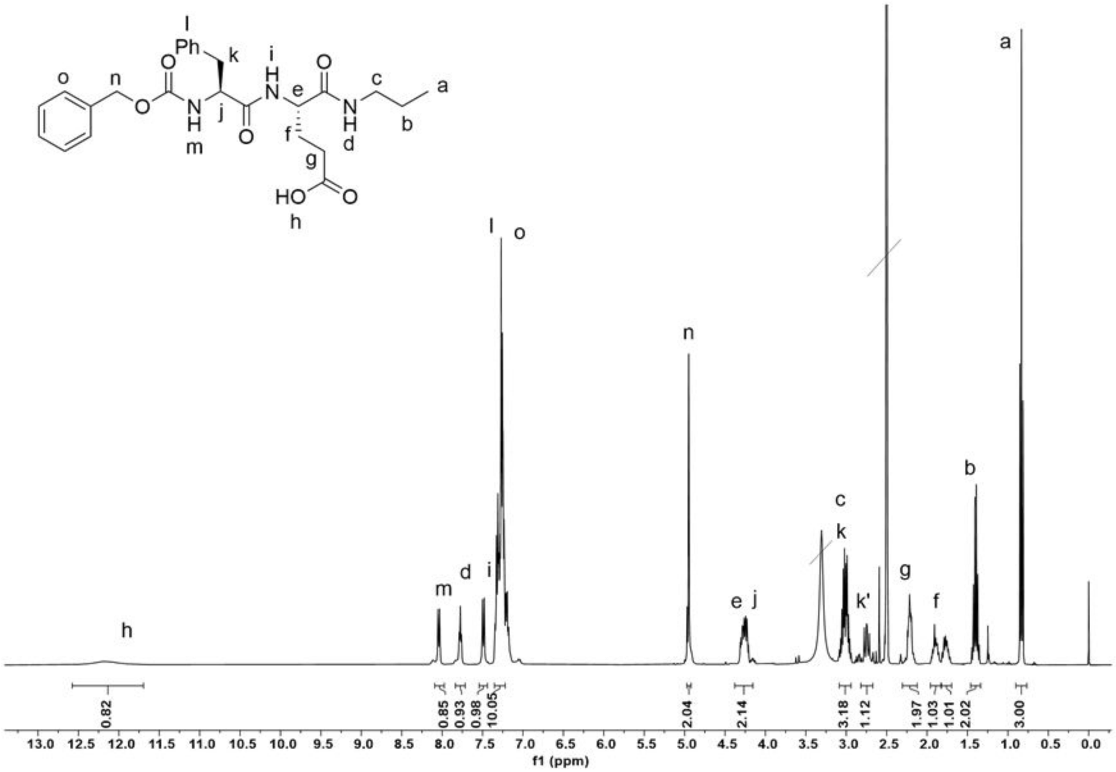

Figure S1. ^{1}H NMR of ZFEC$_3$.

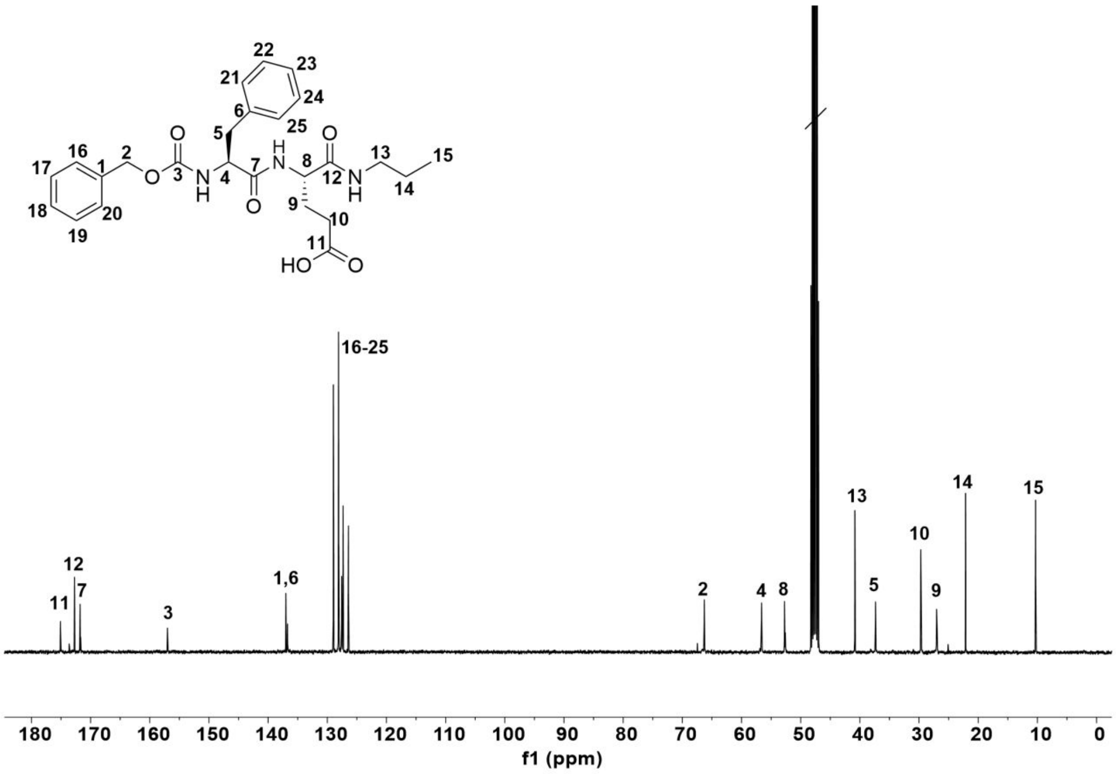

Figure S2. ^{13}C NMR of ZFEC$_3$.

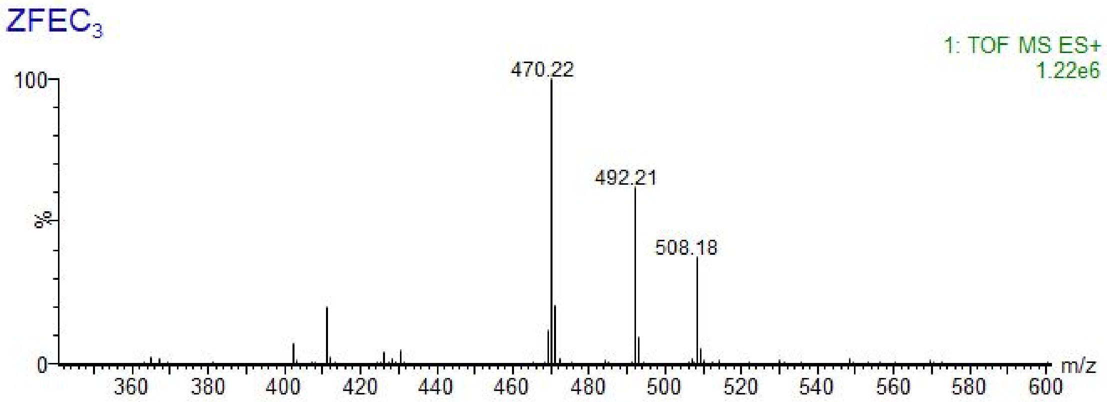

Figure S3. Mass spectrum of $ZFEC_3$ (HRMS (ESI- TOF) m/z).

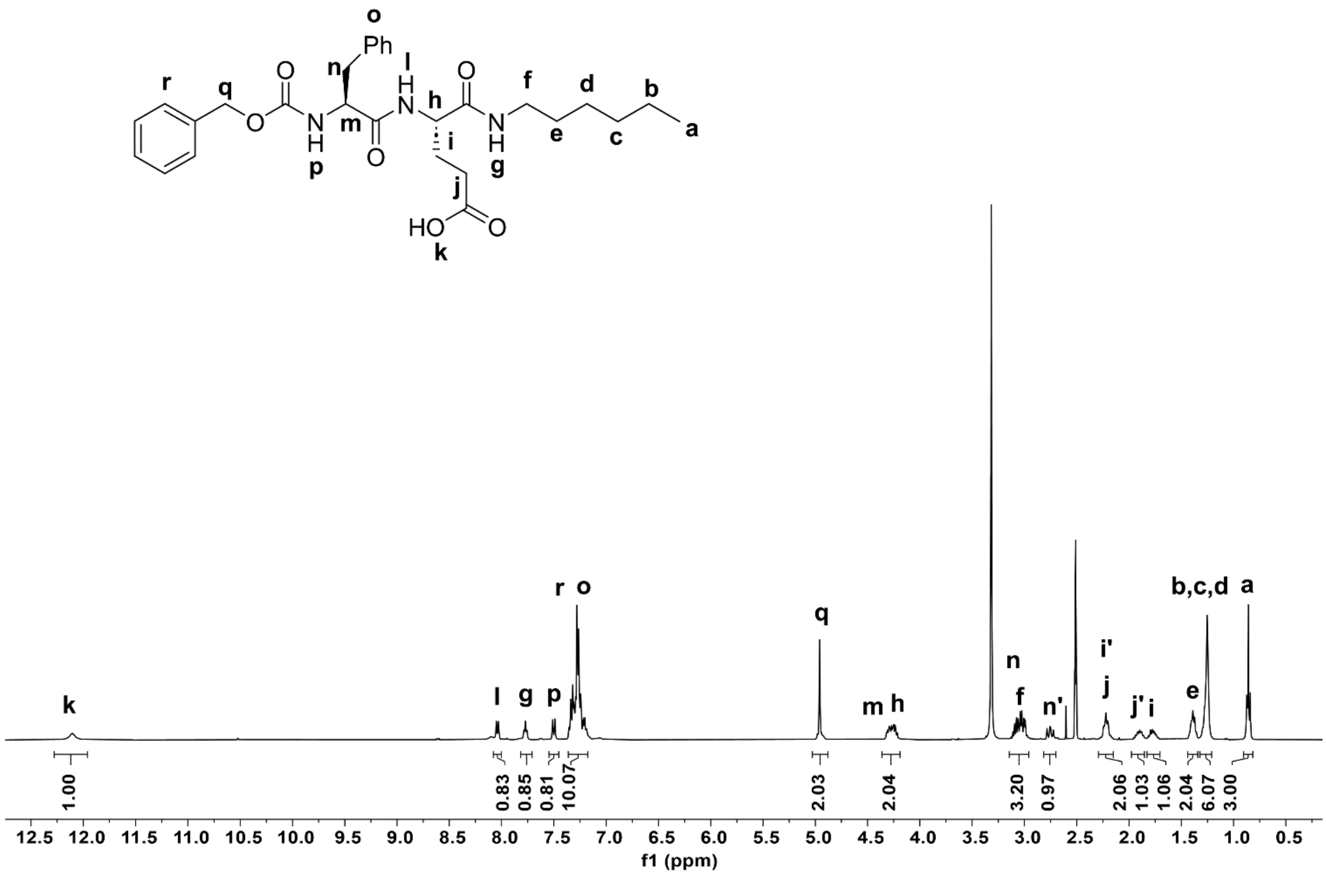

Figure S4. ^1H NMR of $ZFEC_6$.

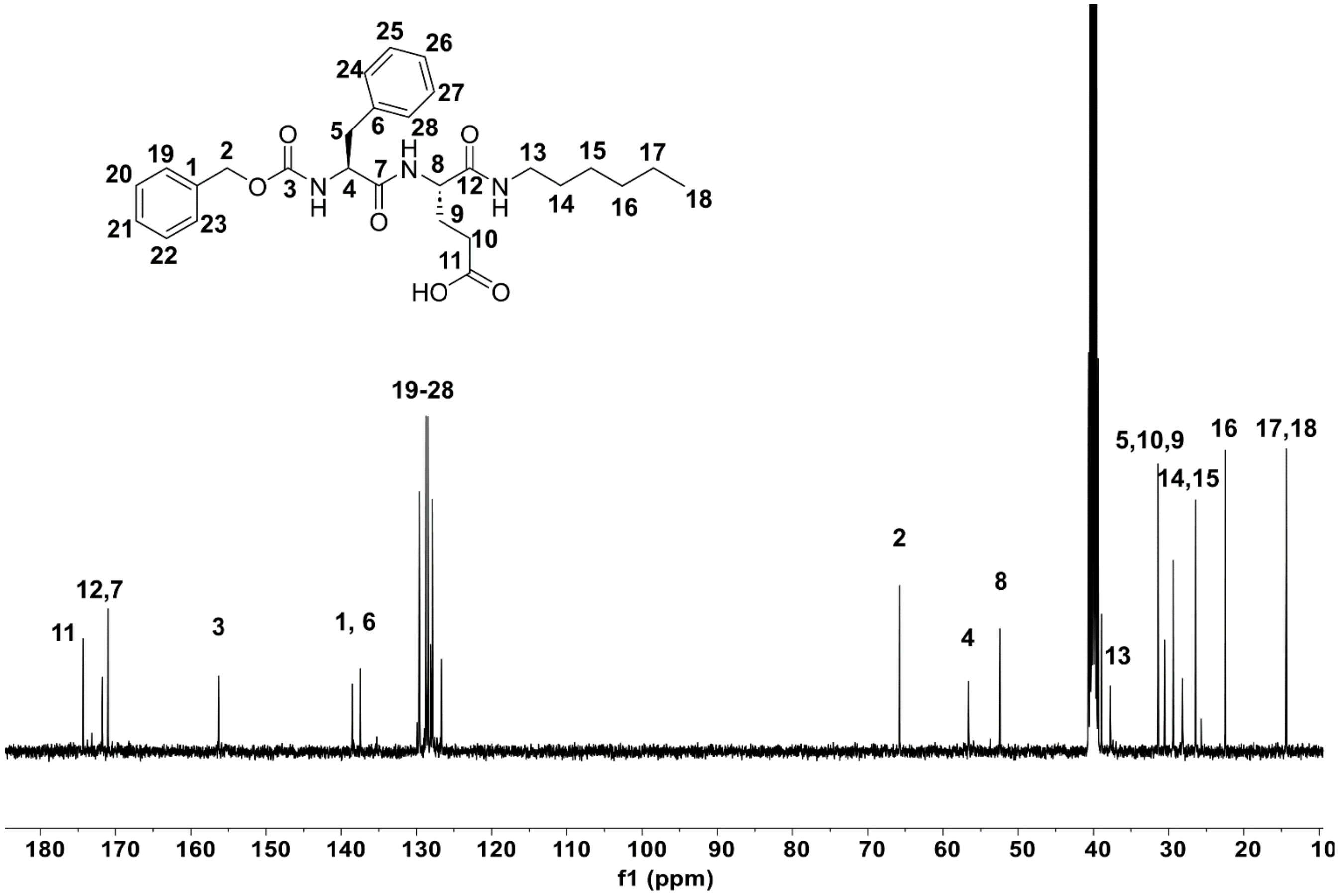

Figure S5. ^{13}C NMR of $ZFEC_6$.

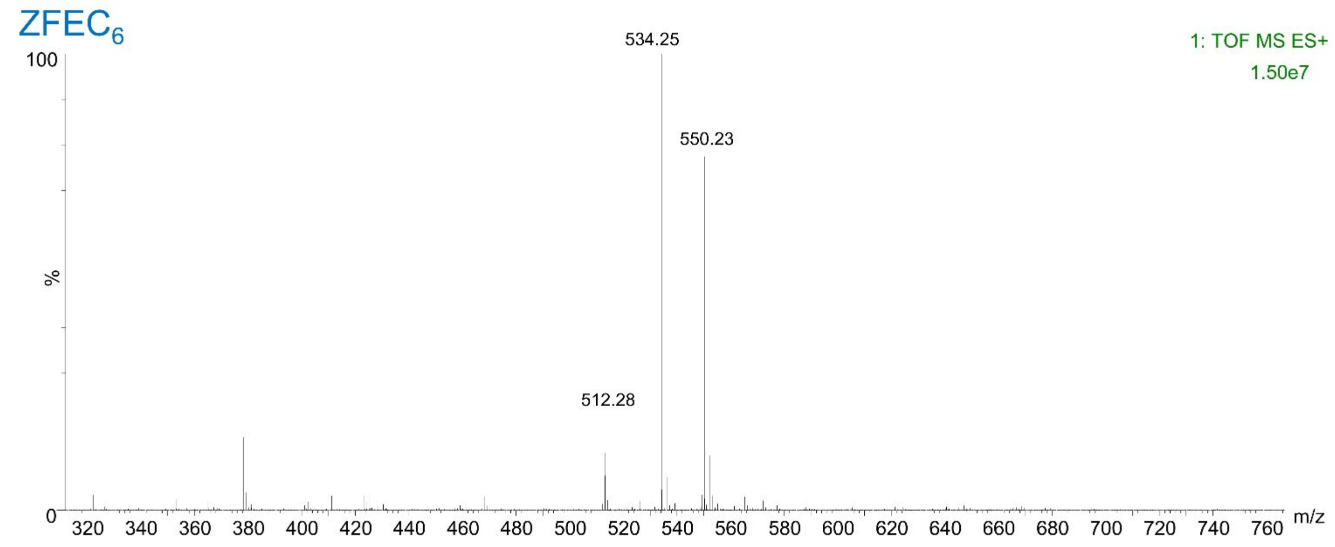

Figure S6. Mass spectrum of $ZFEC_6$ (HRMS (ESI- TOF) m/z).

Synthesis of C3 and C6. The precursor molecules **C3** and **C6** were synthesized by coupling Asp(OtBu)$_2$ to the ZFEC$_3$ / ZFEC$_6$ dissolved in 10 mL DMF. HOBt (2.5 eq.) and HBTU (2.5 eq.) and DIPEA (6 eq.) were added under N_2 at room temperature with stirring for 5 minutes. Di-tert-butyl L-Aspartate Hydrochloride 98.0+% (2.5 eq.) was added and stirred overnight. After removing DMF under vacuum, a mixture (10 mL) of 95% TFA (99%), 2.5% MQ-water, and 2.5% TIPS was added to cleave the tert-butyl protecting group (**Scheme S2**). After removing TFA, purification on RP-HPLC and lyophilizing were performed to get the pure peptide.

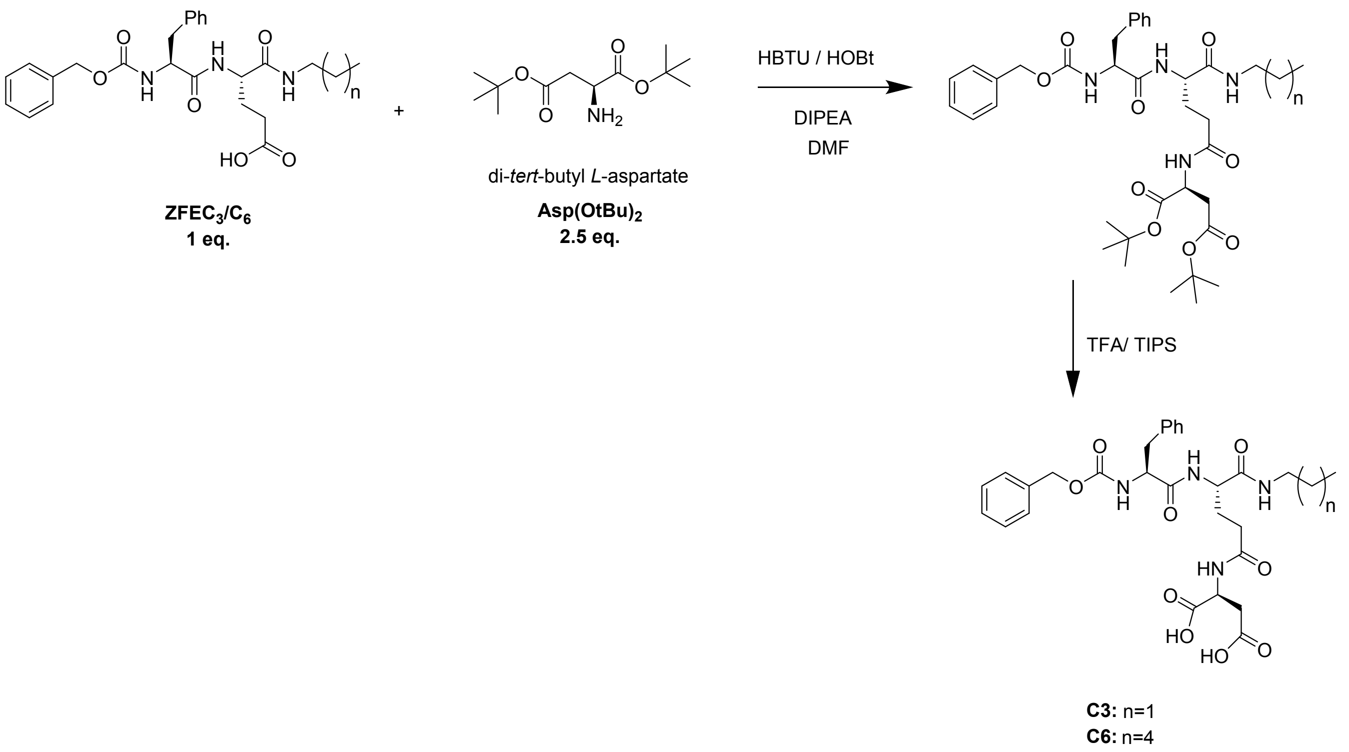

Scheme S2. Synthesis overview of **C3** and **C6.**

Peptide purification. The crude products were purified using reversed-phase High-Performance Liquid Chromatography (RP-HPLC) with gradient elution of water/acetonitrile from 60:40 to 5:95 with 0.1% TFA in MQ-water and flow of 20mL / min. All compounds were detected at 220 nm and 254 nm, lyophilized (Lyophylle: Alpha LDplus, Christ) and characterized by NMR spectroscopy, HPLC, and Electron Spray Ionisation – Mass Spectrometry (ESI-MS).

C3 : ^{1}H NMR (400 MHz, 30 °C, DMSO-d$_6$) δ (ppm) = 12.44 (s, 2H, -COOH; H$_i$), 8.14 (d, *J* = 8.1 Hz, 1H, N-H; H$_h$), 8.08 (d, *J* = 8.0 Hz, 1H, N-H; H$_j$), 7.78 (t, *J* = 5.8 Hz, 1H, N-H; H$_d$), 7.47 (d, *J* = 8.4 Hz, 1H, N-H; H$_n$), 7.38 – 7.17 (m, 10H, Ph-H; H$_m$ and H$_p$), 4.96 (s, 2H, Ph-CH$_2$-O; H$_o$), 4.55 (m,1H, H$_s$), 4.29 (m, 1H, H$_k$), 4.21 (m, 1H, H$_e$), 3.03 (m, 3H, H$_{l'}$ and H$_c$), 2.79 – 2.65 (m, 2H, H$_l$ and H$_{r'}$), 2.62 – 2.55 (m, 1H, H$_r$), 2.24 – 2.05 (m, 2H, H$_g$ and H$_{f'}$), 1.93 – 1.72 (m, 2H, H$_{g'}$ and H$_f$), 1.42 (m, 2H, H$_b$), 0.85 (t, *J* = 7.4 Hz, 3H, H$_a$). ^{13}C NMR (101 MHz, 30 °C, DMSO-d$_6$) δ = 173.0 (C$_{18}$), 172.1 (-COO; C$_{15}$), 171.9 (-COO; C$_{13}$),

171.7 (C_7), 171.2 (C_{16}) , 156.3 (C_3), 138.5 (C_6), 137.5 (C_1), 129.7 - 126.7 (Ph-10C; C_{20-29}), 65.7 (C_2), 56.6 (C_4), 52.8 (C_8), 49.0 (C_{12}), 41.0 (C_{17}), 37.9 (C_5), 36.5 (C_{14}), 32.1 (C_{10}), 28.7 (C_9), 22.7 (C_{18}), 11.8 (C_{19}). ESI-TOF, positive mode: m/z $C_{29}H_{36}N_4O_9$, $[M+H]^+$ = 585.17.

C6 : ^{1}H NMR (400 MHz, 30 ºC, DMSO-d$_6$) δ (ppm) = 13.0-12.1 (s, 2H, , -COOH; H_n), 8.06 (d, J = 8.0 Hz, 2H, N-H; H_o and H_k), 7.75 (t, J = 5.7 Hz, 1H, N-H; H_g), 7.47 (d, J = 8.4 Hz, 1H, N-H; H_t), 7.39 – 7.15 (m, 10H, Ph-H; H_s and H_v), 5.01 – 4.88 (m, 2H, Ph-CH_2-O; H_u), 4.49 (m, 1H, H_l), 4.33 – 4.24 (m, 1H, H_p), 4.24 – 4.14 (m, 1H, H_h), 3.09 – 2.98 (m, 3H, $H_{r'}$ and H_f), 2.74 (m, 1H, $H_{m'}$), 2.63 (m, 1H, H_m), 2.54 (m, 1H, H_r), 2.12 (m, 2H, H_j and $H_{i'}$), 1.84 – 1.78 (m, 2H, $H_{j'}$ and H_i), 1.43 – 1.34 (m, 2H, H_e), 1.33 – 1.15 (m, 6H, H_{b-d}), 0.89 – 0.80 (m, 3H, H_a). ^{13}C NMR (101 MHz, 30 ºC, DMSO-d$_6$) δ (ppm) = 173.9 (C_{16}) , 173.2 (C_{11}), 172.3 (C_{13}), 171.7 (-COO; C_{15}), 171.0 (-COO; C_7), 156.3 (C_3), 138.4 (C_6), 137.4 (C_1) 129.6-127.9 (Ph-10 C; C_{23-32}), 65.8 (C_2) 56.6 (C_4), 52.8 (C_8), 49.0 (C_{12}), 40.7 (C_{17}), 39.0 (C_5), 37.9 (C_{14}), 32.1 (C_{18}), 31.4 (C_{10}), 29.4 (C_{19}), 28.7 (C_9), 26.4 (C_{10}), 22.5 (C_{21}), 14.4 (C_{22}). ESI-TOF, positive mode: m/z $C_{32}H_{42}N_4O_9$, $[M+H]^+$= 627.15.

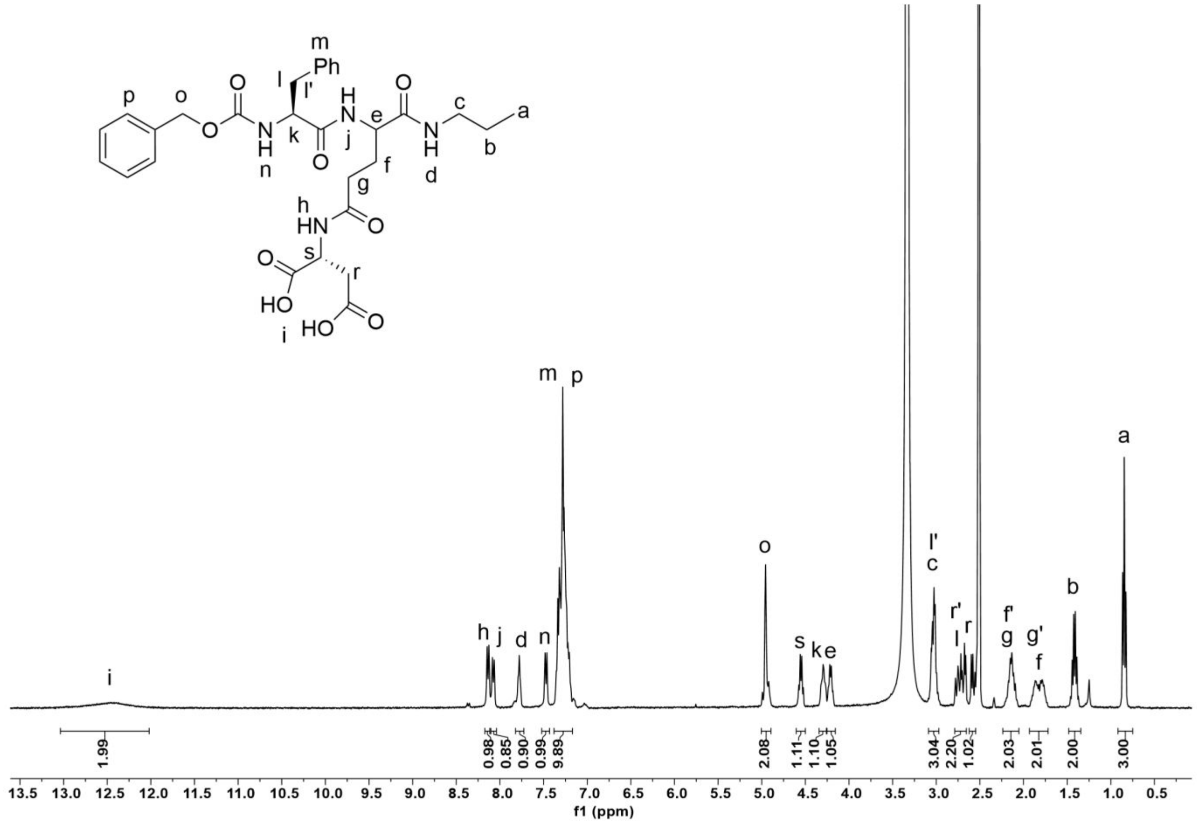

Figure S7. ^{1}H NMR of **C3.**

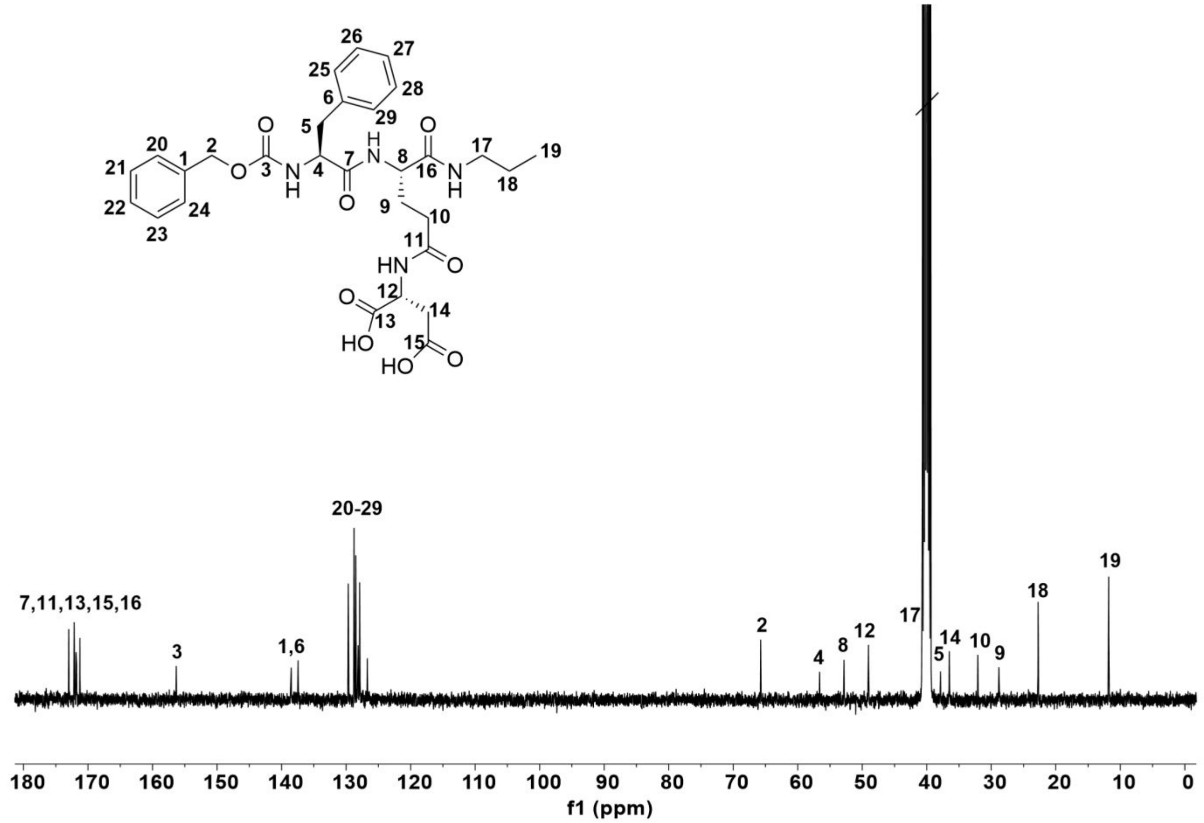

Figure S8. ^{13}C NMR of **C3.**

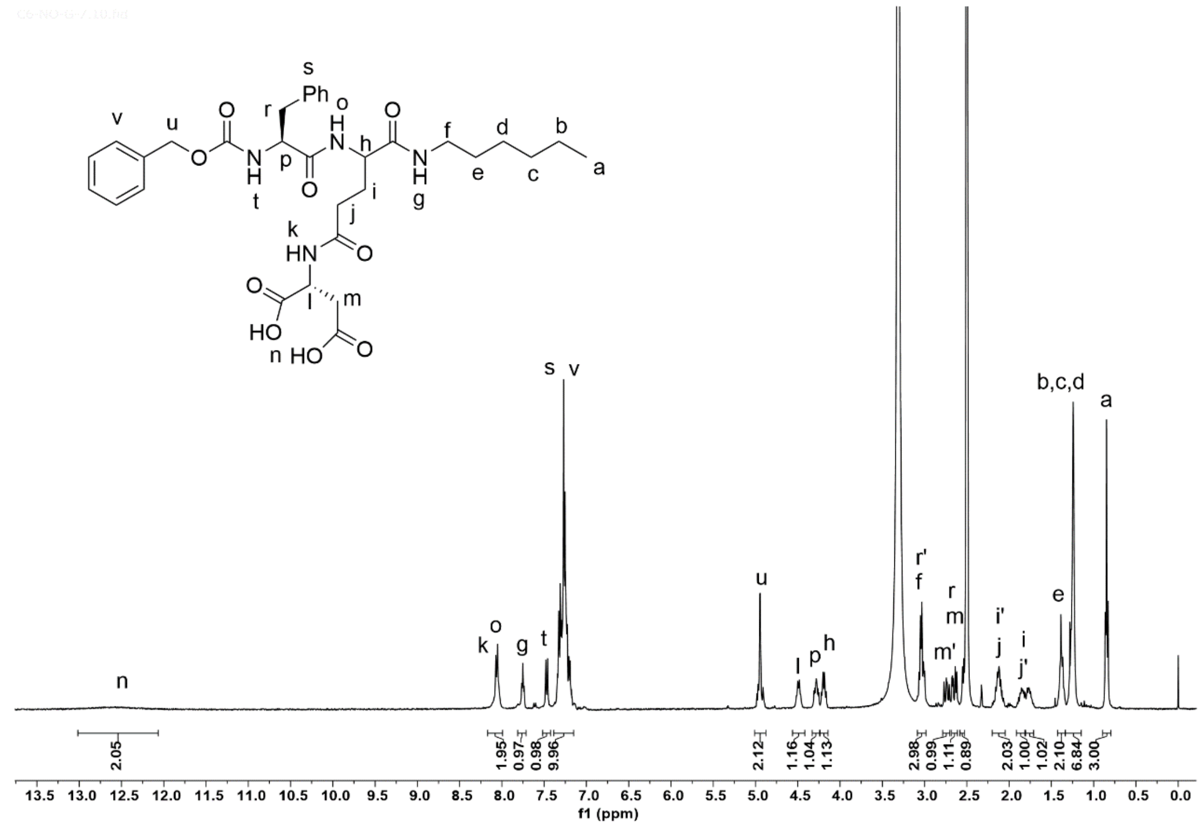

Figure S9. ^{1}H NMR of **C6.**

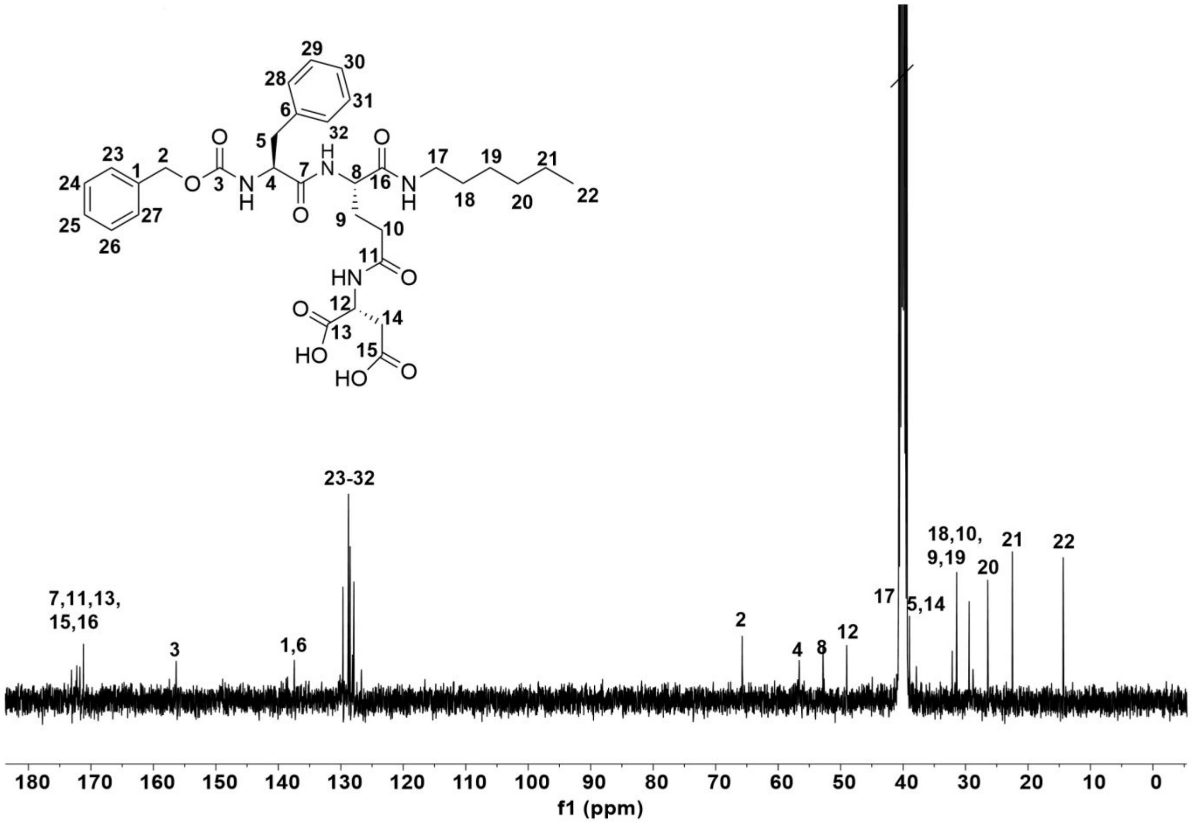

Figure S10. ^{13}C NMR of **C6.**

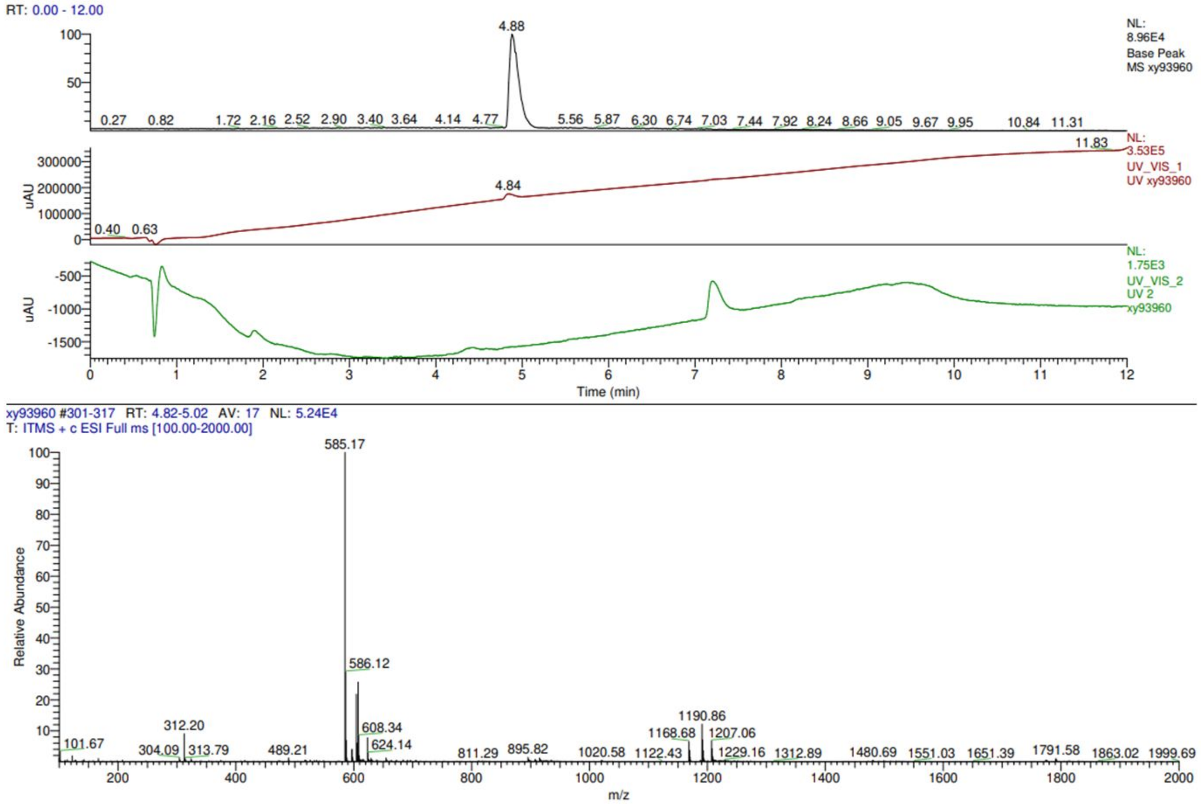

Figure S11. HPLC trace of purified **C3.**

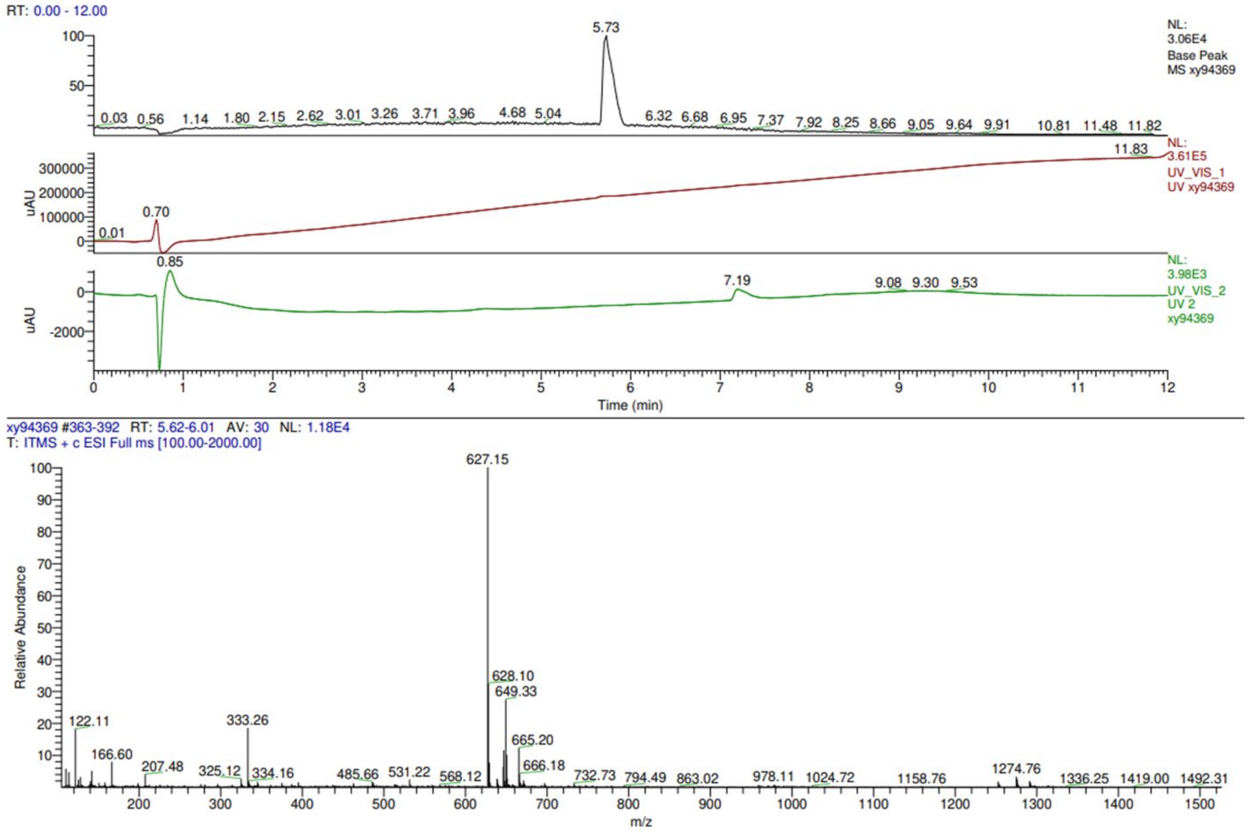

Figure S12. HPLC trace of purified **C6.**

General sample preparation. We used the following standard conditions for all experiments: 2.5 mM in 200 mM MES buffered water, pH 6 at 25 °C, and 15 mM EDC. First, stock solutions of the peptide precursor (2.5 mM) were prepared in 200 mM MES buffer at pH 6. 200 mM MES buffered water was prepared by dissolving the MES hydrate in MQ water. Benzylamine stocks(400 mM) were prepared freshly in acetonitrile. EDC stock solutions were prepared by dissolving the EDC powder in MQ water. Reaction cycles were started by adding EDC from the freshly prepared stock solution to the precursor.

Electron Spray Ionisation – Mass Spectrometry (ESI-MS). A Varian 500 MS LC ion trap or an LCQ Fleet Ion Trap Mass Spectrometer (Thermo Scientific) was used to perform ESI-MS measurements. The samples were diluted in acetonitrile or MQ water before injection into the Mass Spectrometer. All recorded MS data were interpreted using the Thermo Xcalibur Qual Browser 2.2 SP1.48 software.

Potentiometric titration experiments. The titration experiments were conducted by suspending a specified amount of precursor (10 mM) in MQ-water and dissolving it by adding excess base (0.1 M NaOH) to the solution. The titration process was performed by slowly adding small volumes of HCl (0.1 M) at a rate of 0.06 mL/min while simultaneously stirring the solution using a magnetic stirrer. The titration curves were analyzed with the Hyperquad2013 and HYSS2009[2] programs to obtain the respective apparent pKa of the peptide precursor.

Analysis of the reaction kinetics by HPLC. The kinetics of the chemical reaction cycles were monitored over time by analytical HPLC (ThermoFisher Vanquish Duo UHPLC, a Hypersil Gold 100 x 2.1 mm C18 column (3 µm pore size). After starting the reaction cycle by adding EDC to the peptide precursor solution with a total volume of 10 µL, 10 µL of 400 mM benzylamine were added at certain time points. After 24 hours, the resulting quenched clear solution was measured via HPLC to determine the EDC, guanidine and benzylamide concentrations. The ratio of the reaction solution and benzylamine solution (400 mM) was 1:1. 0.5 µL quenched solution was directly injected without further dilution into HPLC and tracked with a UV/Vis detector at 220 and 254 nm. All compounds involved were separated using a method of a linear gradient of H2O: ACN, each with 0.1% TFA. Measurements were performed in triplicate (n = 3) at 25°C.

Determination of the assembled peptide concentration during the reaction cycle. ^{1}H-NMR spectra were conducted on a Varian Inova 300 (300 MHz). Chemical shifts are reported as δ-values in parts per million (ppm) relative to the internal standard hydroquinone (50 mM) in D_2O (δH: 6.70) or the deuterated solvent peak: DMSO-d_6 (δH: 2.50). An inner tube with an internal hydroquinone standard (50 mM) dissolved in D_2O was used. The ^{1}H-NMR spectra were recorded with water suppression. The non-assembled peptide concentration was determined by comparing the integral of the protecting group- protons at 7.20 – 7.50 ppm to the integral of the internal hydroquinone standard at 6.70 ppm.

Confocal Fluorescence Microscopy. Confocal fluorescence microscopy was performed on a Leica TCS SP8 confocal microscope using a 63x oil/water immersion objective with a numerical aperture of 1.52. Samples with a total reaction volume of 30 µL were prepared directly in an ibidi µ-slide angiogenesis well plate with sterilized glass-bottom. 5 µM Nile red was added as a dye with excitation at 552 nm and emission at 577 – 656 nm. Imaging was conducted at 22 °C.

Turbidity measurements. Turbidity measurements were carried out at on a Microplate Spectrophotometer (Thermo Scientific Multiskan GO, Thermo Scientific SkanIt Software 6.0.1). Measurements were performed in a non-tissue culture treated 96-well plate (Falcon, flat bottom). Every 30 seconds, the absorbance of the 100µL samples was measured at 600 nm. All experiments were performed in triplicate (n = 3) at 25 °C.

Nile Red assay. 2.5 µM Nile Red was added to the reaction solution of 2.5 mM peptide with 15 mM EDC. The fluorescence intensities were measured at 1h and 12h at emission wavelengths of 575 – 800 nm and excitation at 550 nm. The excitation and emission slit width was 12 nm.

Transmission Electron Microscopy. Samples were applied directly onto Carbon support film on 200 mesh copper grids. Transmission electron microscopy (TEM) images were recorded in a JEM 2100plus Transmission Electron Microscope.

Scanning Electron Microscopy. Previously prepared transmission electron microscopy (TEM) grids were subsequently positioned atop an aluminum specimen mount stub and coated with platinum

through sputtering. Afterward, the scanning electron microscopy (SEM) analysis was executed utilizing a Leica-Zeiss LEO 440 microscope.

ATR-FTIR. Prepared samples were lyophilized and FT-IR spectra were collected in a JASCO FTIR-6200 spectrometer - ATR PRO ONE at a wavenumber range of 400-4000 cm^{-1}.

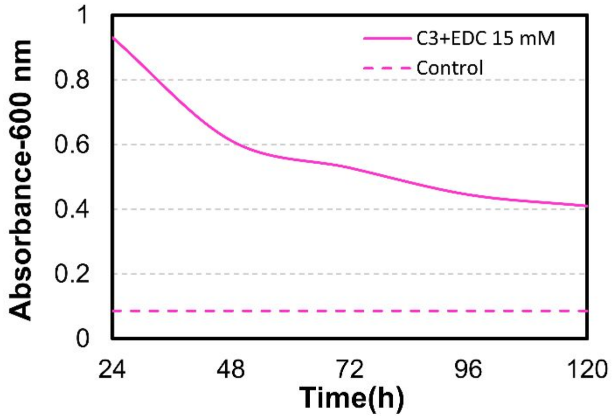

Figure S13. To assess the dynamics of the assembly and disassembly process, the turbidity of the solution of **C3** in response to EDC was followed in a plate reader every 24h up to 120h. We observed a certain decrease on absorbance value, which indicates the anhydride hydrolysis by time.

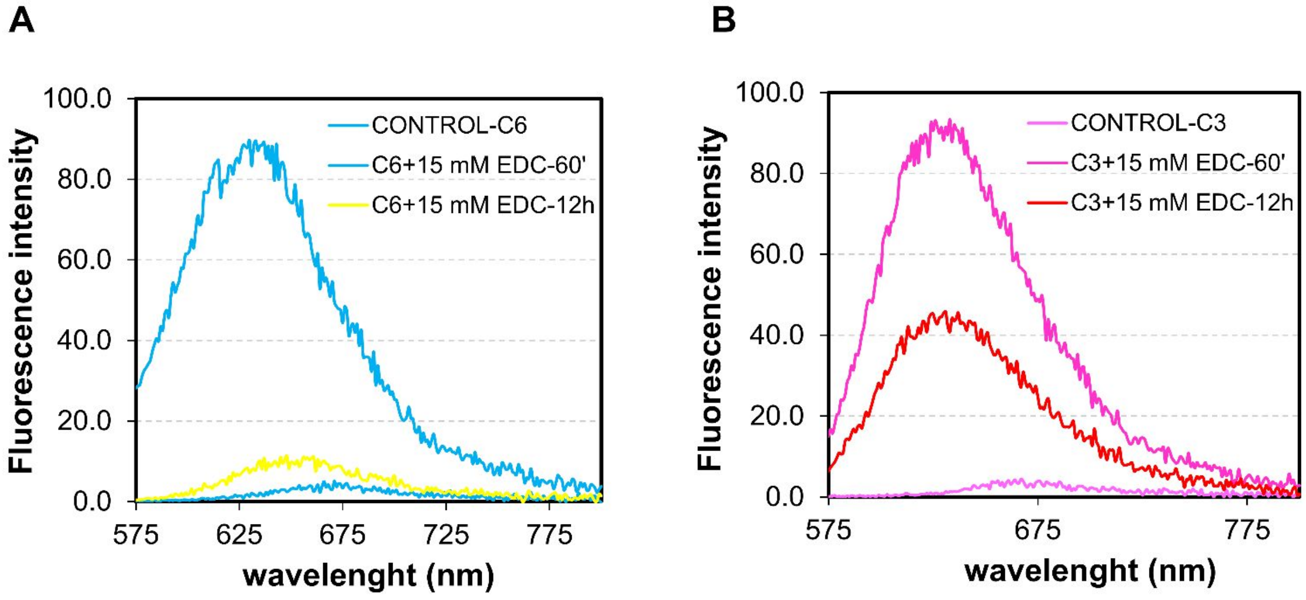

Figure S14. C6 (**A**), and **C3** (**B**) with 15 mM EDC, stained with Nile Red dye. The fluorescence intensity was measured at 1 hour and 12 hours, using emission wavelengths ranging from 575 to 800 nm (λ_{ex} =550 nm).

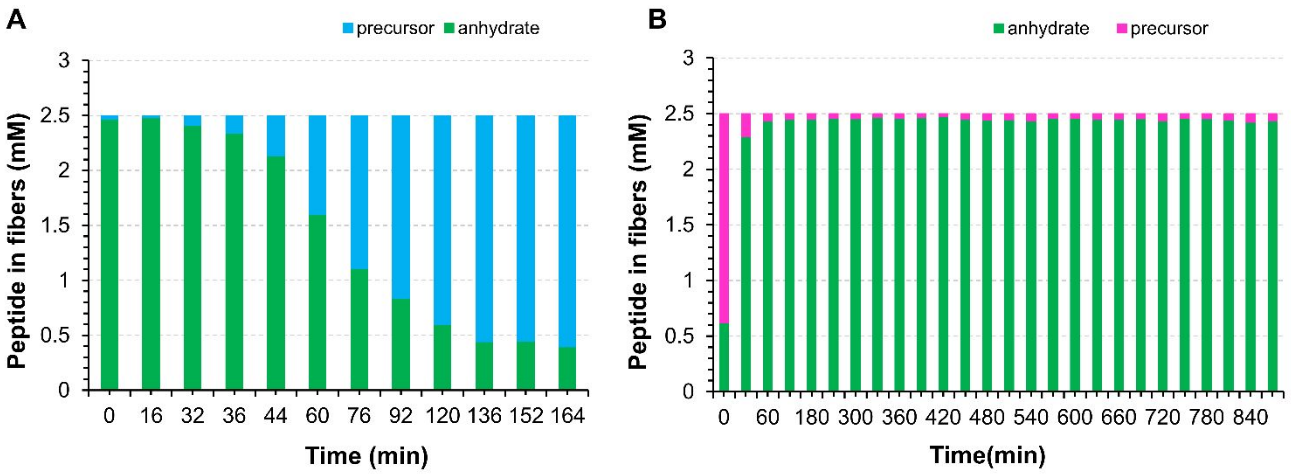

Figure S15. The time-dependent concentrations of precursor and anhydride product in the assemblies were determined by using ^{1}H-NMR spectroscopy. Assembled precursor concentration was calculated by assuming all anhydride is assembled, using the determined assembled peptide concentration and anhydride concentration. The experiment was conducted using 2.5 mM **C6 (A)** and 2.5 mM **C3 (B)** fueled with 15 mM EDC, in 200 mM MES buffer, pH 6.

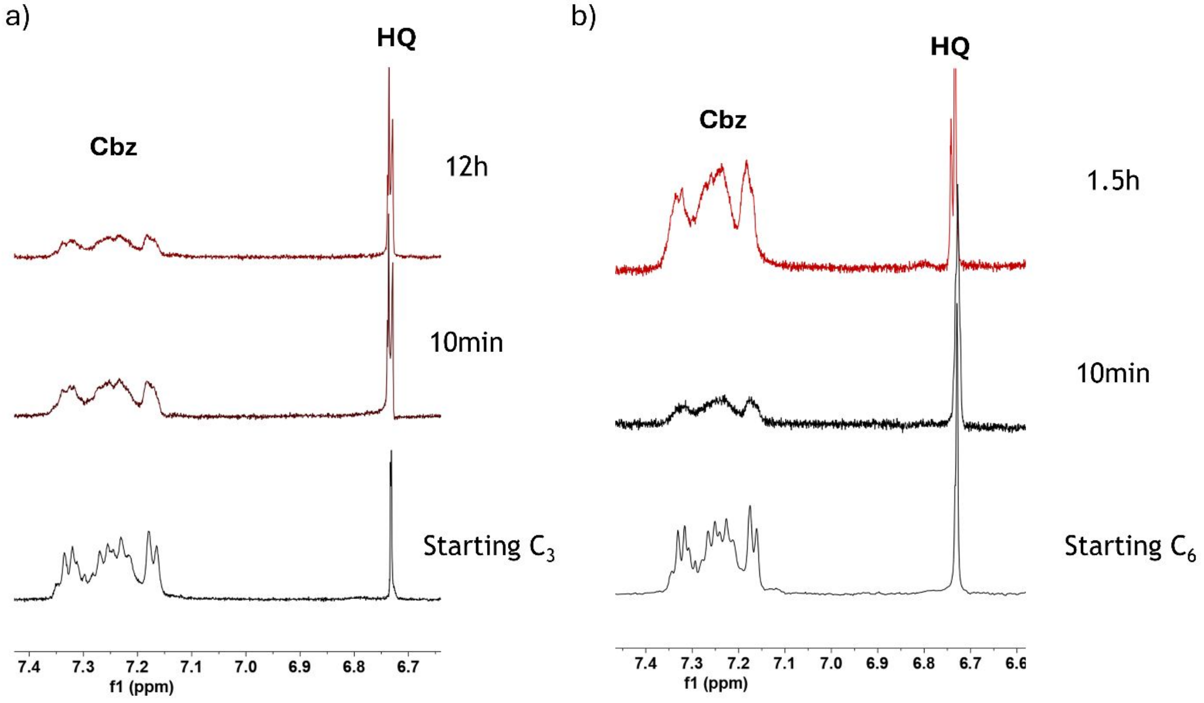

Figure S16. Selected ^{1}H NMR spectra used to estimate peptide assembly concentrations for a)**C3** and b) **C6** at representative time points after EDC fueling.

Freely dissolved peptides give sharp ^{1}H NMR signals, whereas peptides incorporated into supramolecular assemblies are NMR silent due to restricted molecular motion. Thus, signal attenuation directly reflects the fraction of peptide in the assembled (activated) state. The activated peptide concentration at each time point was determined by subtracting the concentration of free peptide from the total peptide concentration (2.5 mM). The free peptide concentration was obtained by normalizing the CBz aromatic proton integral to an internal standard and referencing this ratio to the value at time zero.

Using this approach, **C3** showed ~1.7 mM activated peptide at 10 min, increasing to ~2.36 mM at 12 h. In contrast, **C6** exhibited ~2.41 mM activated peptide at 10 min, followed by a decrease to ~1.16 mM at 1.5 h. These results are consistent with turbidity measurements indicating more rapid disassembly for **C6** than for **C3**.

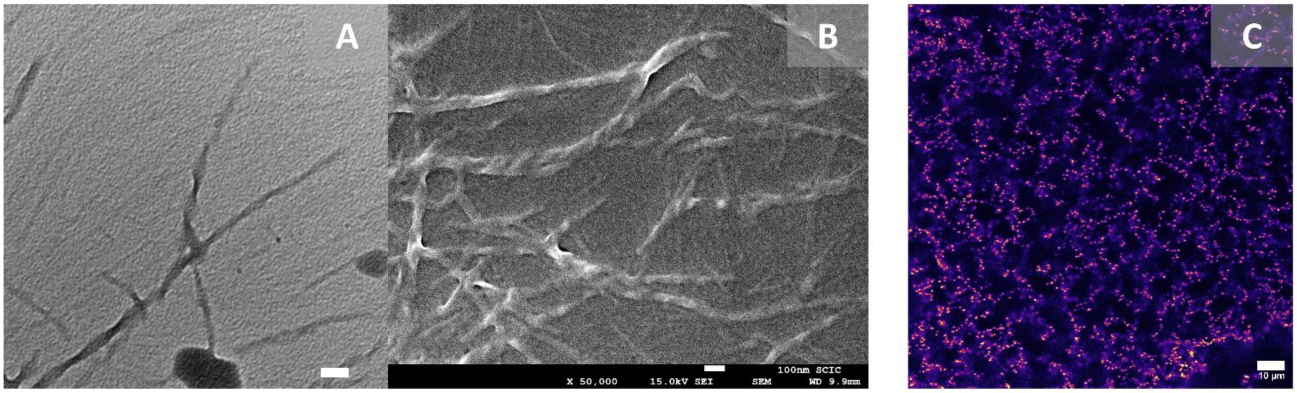

Figure S17. Micrographs of **C6** were captured by TEM (A) and SEM (B) (scale bars = 100 nm) and by confocal microscopy (C) (scale bar = 10 µm) 30 min after EDC fueling.

The twisted nanostructure observed for **C6** likely reflect a later, more developed / mature stage of the assembly formed after EDC fueling.

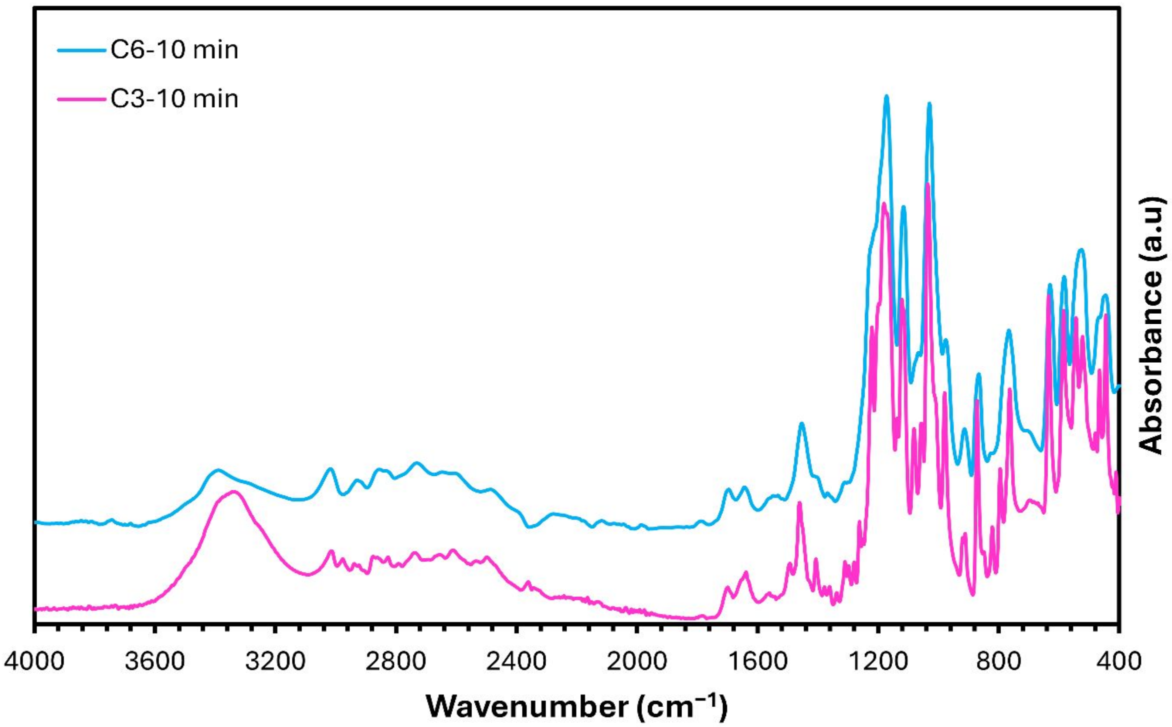

Figure S18. Full ATR–FTIR spectra of **C3** and **C6** at 10 min after fueling with EDC.

The full ATR–FTIR spectra of **C3** and **C6** collected 10 min after fueling with EDC are shown in Figure S17. Bands observed in the 1550-1450 cm^{-1} and 1400-1300 cm^{-1} regions are consistent with carboxylate stretching modes of Asp residues. Compared to **C3**, **C6** exhibits broader and more intense features in these regions, indicating differences in the local environment and intermolecular interactions of the carboxylate groups. No bands characteristic of anhydride carbonyl stretching (~1820 and ~1760 cm^{-1}) are observed, suggesting that any anhydride species formed are transient and present at low steady-state levels.

2. Computational Methods

System setup. The monomers **C3** and **C6** were manually modelled using Pymol[3] software, the geometry was cleaned and then the four possible ß-sheets (**C3** / **C6** -parallel, **C3** / **C6** -antiparallel) were built using 12 monomers in each case. This central ß-sheet was wrapped by four chains of 10 monomers also arranged in a ß-sheet conformation, so each system was formed by 52 monomers. In order to do the molecular dynamic (MD) simulations the missing force field (FF) parameters of the carbobenzoxy (CBZ), anhydride ring residue (ANH), and the alkyl amide chain (CHN) were computed using General Amber Force Field (GAFF)[4] , and the atomic charges were computed through the AM1 method with bond charge corrections (AM1-BCC)[5] using the Antechamber software[6] (**Table S1**), which is available in the AmberTools package. Afterwards, the complexes were solved in a water box keeping

a distance of 10 Å between the outer monomers and the edges of the box using tleap program.[7] The water molecules were treated using TIP3P FF.[8] The all systems had between 46000 and 48000 atoms.

Molecular Mechanics/Molecular dynamics simulations. The first step was the minimization of the system, doing 10^5 steps by means of the conjugate gradient algorithm, followed by MM/MD simulations. NAMD software[9] was used for all calculations using a NVT ensemble with AMBER ff03,[10] a force field suitable for proteins but also for these peptide-like aggregates. For the minimization of the positions to form intermolecular hydrogen bonds, expected in a parallel and an antiparallel arrangement for both monomers, were constrained (3 A and 500 $kcal \cdot mol^{-1} \cdot Å^{-2}$) to let the lateral chains to adapt to this conformation. All models were heated to the temperature of the experiment (298 K), keeping the constraints. This was possible through a short MD simulations of 10 ps using a constant increment of 0.1 K/fs. Then the constrained system was equilibrated through a 100 ps NPT MD at 298 K using a Langevin piston and thermostat for pressure and temperature control.[11] The last frame of this trajectory was saved, and subsequently, 50 ns of NVT MD were carried out with the same constraints as before. Lastly, 40 ns NVT MD simulations were carried out, releasing the central ß-sheet to study conformational stability. When the unbiased system was equilibrated (**Figure S21**), i.e., the RMSD of the central chain showed little variation, the analysis of intermolecular interactions was carried out. In order to do it, only the last 10 ns were analyzed.

Periodic boundary conditions (PBC) were applied to all simulations, and the selected time step was 1 fs. To account for non-bonding interactions, the Particle Mesh Ewald (PME) algorithm[12] is used with a smooth switching function at a distance from 14.5 to 16 Å. The results were analyzed using to cpptraj program[13] of AmberTools (**see Results and Discussion** section).

Interaction energies. The electrostatic and van der Waals interaction energies were computed using the linear interaction energy (LIE) method[14] for each frame from the last 10ns of MD simulations. The non-bonded interactions between all atoms of the selected residue/chain fragment and surroundings were obtained using the cpptaj program. The electrostatic interactions were calculated according to a shifting function:

$$E_{elec} = \frac{kq_iq_j}{r_{ij}}\left(1 - \frac{r_{ij}^2}{r_{cut}^2}\right)$$

where the k is Coulomb's constant, q_i is a total charge of the selected fragment, q_j is a total charge of the surrounding region, r_{ij} s the distance between the charge centers and r_{cut} is the cut-off distance, set to 8 Å.

The vdW interactions are computed using the 12-6 Lennard-Jones potential according to:

$$E_{vdW} = 4\varepsilon_{ij}\left[\left(\frac{\sigma_{ij}}{r_{ij}}\right)^{12} - \left(\frac{\sigma_{ij}}{r_{ij}}\right)^{6}\right]\left(1 - \frac{r_{ij}^2}{r_{cut}^2}\right)$$

where r_{ij} is the interatomic distance, σ_{ij} is the distance at which the LJ potential is zero, i.e., where attraction and repulsion balance out, and ε is the depth of the potential well, representing the strength of the interaction.

Total interaction energies within a distance of 8 Å were obtained as a sum of electrostatic and van der Waals contributions.

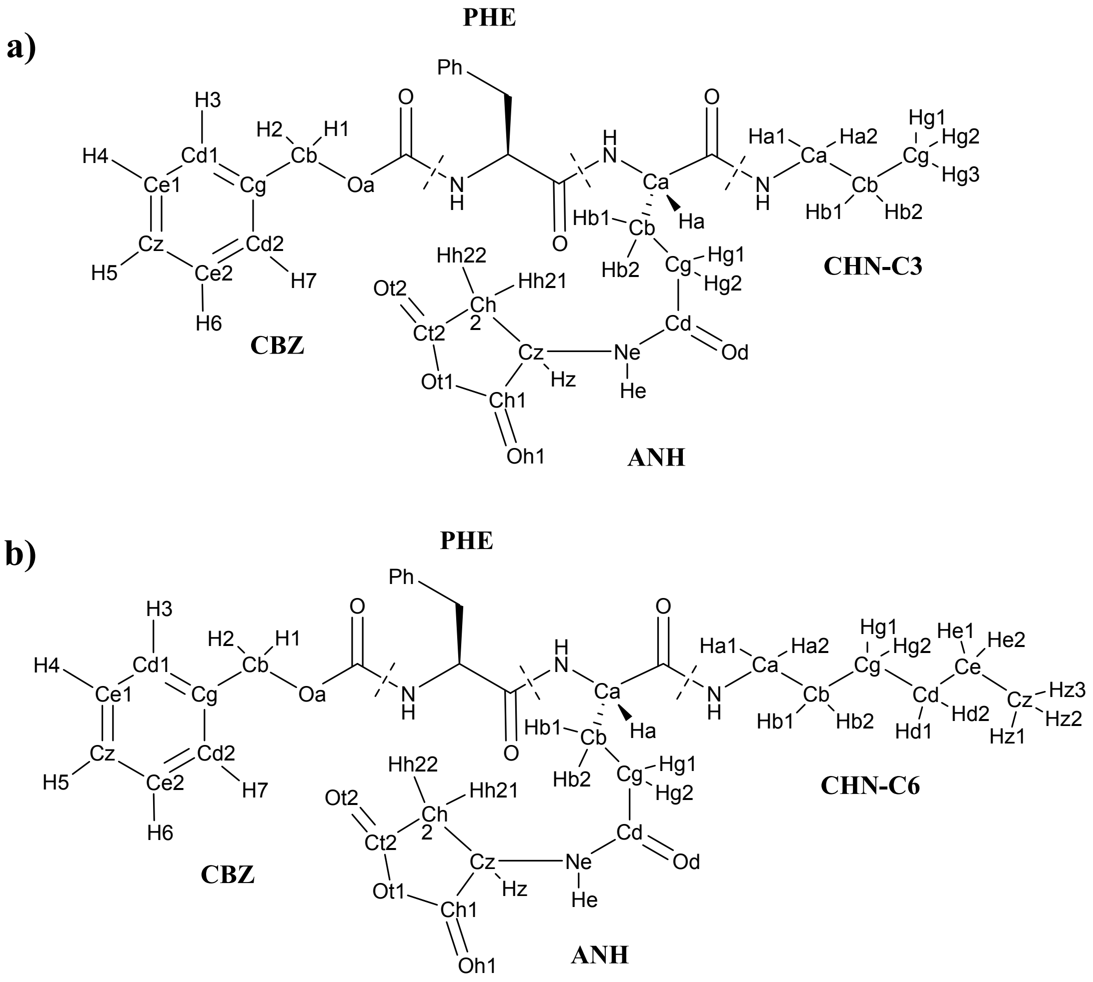

Figure S19. Numbering of the atoms of **a)** monomer **C3,** and **b)** monomer **C6.**

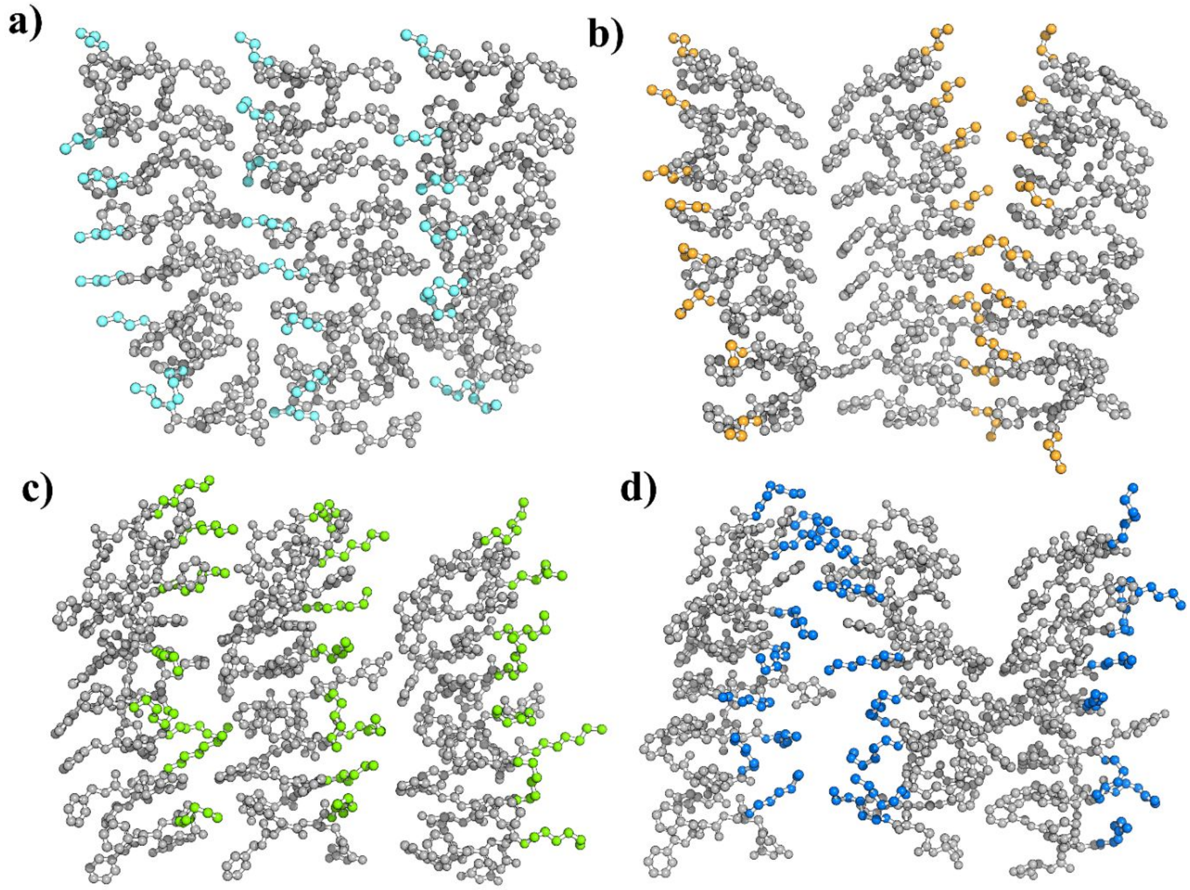

Figure S20. Snapshot of the arrangement of the three chains for the systems built for the study of the interphase through MD simulations, with the aliphatic chains highlighted: **C3** (a) and **C6**-parallel (c) alkyl chains in light blue and green, **C3** (b) and **C6**-flipped (d) alkyl chains in orange and marine blue, respectively.

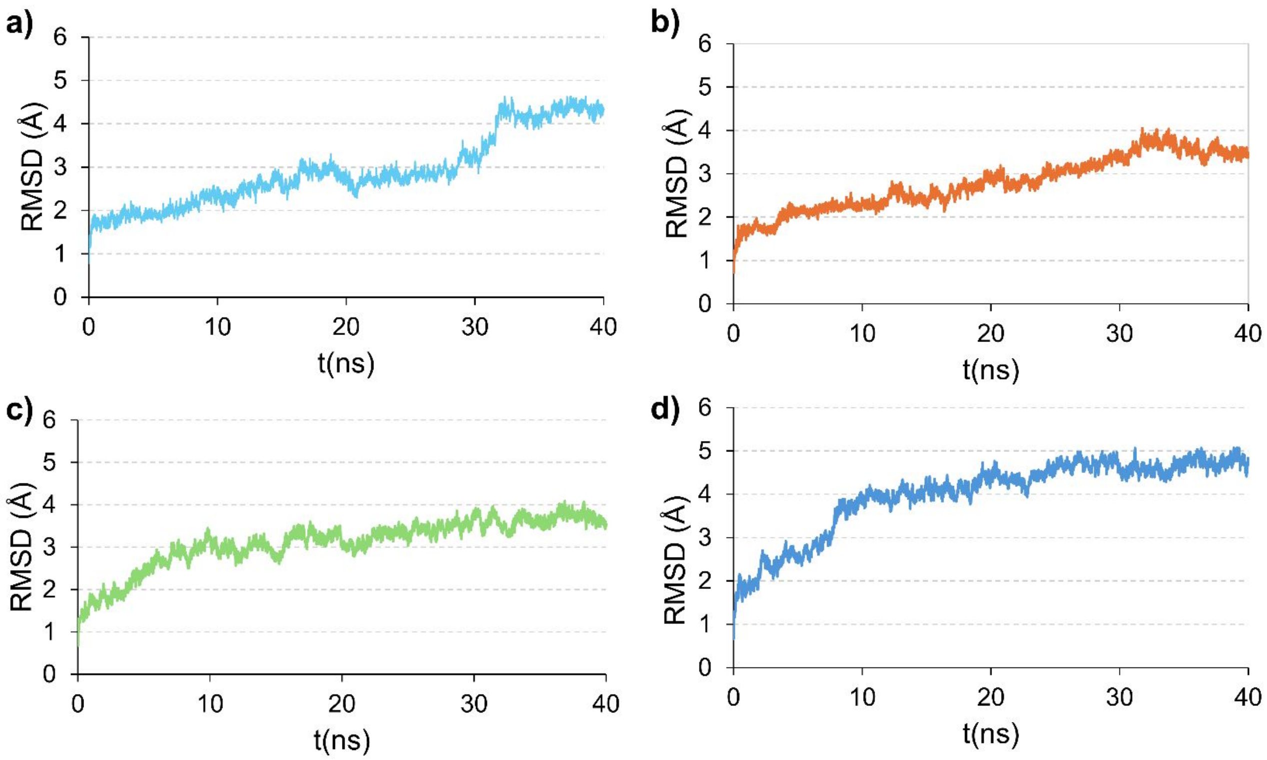

Figure S21. RMSD of the central β-sheet with **a**) **C3** -parallel, **b**) **C3** -antiparallel, **c**) **C6** -parallel and **d**) **C6** -antiparallel arrangement.

Table S1. Atom types, charges and parameters for bonding and non-bonding interactions obtained for CBZ, ANH, CHN-C3 and CHN-C6 of both inhibitors computed using Antechamber software.[15]

Cbz group					
**Atom name**	**Atom type**	**Charge**	**Atom name**	**Atom type**	**Charge**
Oα	os	-0.432194	H6	ha	0.136215
Cβ	c3	0.184044	H5	ha	0.135191
Cγ	ca	-0.122271	H4	ha	0.136215
Cδ1	ca	-0.105389	H3	ha	0.146457
Cε1	ca	-0.132713	H1	h1	0.072409
Cζ	ca	-0.116123	H2	h1	0.072409
Cε2	ca	-0.132713	C	c	0.649223
Cδ2	ca	-0.105389	O	o	-0.531826
H7	ha	0.146457			
Parameters:					
**Mass**			**Bond**		
os	16	0.465	c3-os	308.6	1.432
c3	12.01	0.878	c-os	390.8	1.358

ca	12.01	0.36	c3-ca	321	1.516
ha	1.008	0.135	c3-h1	330.6	1.097
h1	1.008	0.135	ca-ca	461.1	1.398
c	12.01	0.616	ca-ha	345.8	1.086
o	16	0.434	c-o	637.7	1.218
			c-N	427.6	1.379
**Angle**			**Angle**		
ca-c3-os	68.3	108.95	ca-ca-ha	48.2	119.88
h1-c3-os	50.8	109.78	h1-c3-h1	39.2	108.46
o-c-os	75.3	123.25	N-c-o	74.2	123.05
c-os-c3	63.3	115.98	c-N-H	48.3	117.55
c3-ca-ca	63.5	120.77	c-N-CT	63.4	120.69
ca-c3-h1	47	109.56	N-c-os	74.3	112.82
ca-ca-ca	66.6	120.02			
**Dihedrals**					
os-c3-ca-ca		6	0	0	2
o-c-os-c3		1	2.7	180	-2
o-c-os-c3		1	1.4	180	1
c3-ca-ca-ca		4	14.5	180	2
c3-ca-ca-ha		4	14.5	180	2
ca-ca-ca-ca		4	14.5	180	2
ca-ca-ca-ha		4	14.5	180	2
ha-ca-ca-ha		4	14.5	180	2
h1-c3-ca-ca		6	0	0	2
ca-c3-os-c		3	1.15	0	3
h1-c3-os-c		3	1.15	0	3
o-c-N-H		1	2.5	180	-2
o-c-N-H		1	2	0	1
o-c-N-CT		4	10	180	2
os-c-N-H		4	10	180	2
os-c-N-CT		4	10	180	2
**Impropers**					
c3-ca-ca-ca		1.1	180	2	
ca-ca-ca-ha		1.1	180	2	
**Nonbonding**			**Nonbonding**		
os	1.6837	0.17	h1	1.387	0.0157
c3	1.908	0.1094	c	1.908	0.086
ca	1.908	0.086	o	1.6612	0.21
ha	1.459	0.015			

<u>**Anh group**</u>					
**Atom name**	**Atom type**	**Charge**	**Atom name**	**Atom type**	**Charge**

N	n	-0.5669	Cτ2	c	0.742236
HN	hn	0.372159	CH2	c3	-0.194941
Cα	c3	0.045309	HH21	hc	0.1127
Cβ	c3	-0.073223	HH22	hc	0.1127
Hβ2	hc	0.076765	Oτ2	o	-0.494061
Hβ1	hc	0.076765	OH1	o	-0.484477
Cγ	c3	-0.142228	Hζ	h1	0.108013
Hγ1	hc	0.069995	HNε	hn	0.350494
Cδ	c	0.691719	Oδ	o	-0.560767
Nε	n	-0.546199	Hγ2	hc	0.069995
Cζ	c3	0.047601	Hα	h1	0.114262
CH1	c	0.716196	C	C	0.547771
Oτ1	os	-0.407517	O	O	-0.505179
<u>Parameters:</u>					
**Mass**			**Bond**		
n	14.01	0.53	hn-n	403.2	1.013
hn	1.008	0.161	c3-n	328.7	1.462
c3	12.01	0.878	c3-c3	300.9	1.538
hc	1.008	0.135	c3-h1	330.6	1.097
c	12.01	0.616	c-c3	313	1.524
os	16	0.465	c3-hc	330.6	1.097
o	16	0.434	c-n	427.6	1.379
h1	1.008	0.135	c-o	637.7	1.218
			c-os	390.8	1.358
			C-n	427.6	1.379
**Angle**			**Angle**		
c3-c3-n	65.9	111.61	c-n-c3	63.4	120.69
h1-c3-n	49.8	108.88	c-n-hn	48.3	117.55
c-c3-n	67	109.06	n-c-o	74.2	123.05
c3-n-hn	45.8	117.68	c3-c-os	68.9	110.72
c3-c3-hc	46.3	109.8	c-c3-h1	47	108.22
c3-c3-c3	62.9	111.51	c-os-c	63.8	120.64
c3-c-o	67.4	123.2	o-c-os	75.3	123.25
c3-c3-h1	46.4	109.56	n-C-O	74.2	123.05
c-c3-c3	63.3	111.04	C-n-hn	48.3	117.55
hc-c3-hc	39.4	107.58	C-n-c3	74.3	112.82
c3-c-n	66.8	115.18	CT-C-n	63.4	120.69
c-c3-hc	46.9	108.77			
**Dihedrals**					
hc-c3-c3-n		9	1.4	0	3
c3-c3-c3-n		9	1.4	0	3
o-c-c3-n		6	0	180	2
c3-c3-n-hn		6	0	0	2
h1-c3-n-hn		6	0	0	2

c-c3-n-hn	6	0	0	2
c3-c3-c3-hc	1	0.16	0	3
c-c3-c3-c3	9	1.4	0	3
o-c-c3-c3	6	0	180	2
n-c-c3-c3	1	0.1	0	-4
n-c-c3-c3	1	0.07	0	2
hc-c3-c3-hc	1	0.15	0	3
c-c3-c3-hc	9	1.4	0	3
c3-c-n-c3	1	0	0	-2
c3-c-n-c3	1	1.5	180	1
c3-c-n-hn	4	10	180	2
n-c-c3-hc	6	0	180	2
o-c-c3-hc	1	0.8	0	-1
o-c-c3-hc	1	0	0	-2
o-c-c3-hc	1	0.08	180	3
c-c3-n-c	1	0.85	180	-2
c-c3-n-c	1	0.8	0	1
c3-c3-n-c	1	0.5	180	-4
c3-c3-n-c	1	0.15	180	-3
c3-c3-n-c	1	0	0	-2
c3-c3-n-c	1	0.53	0	1
h1-c3-n-c	6	0	0	2
os-c-c3-n	6	0	180	2
c-c3-c3-n	9	1.4	0	3
c3-c-os-c	2	5.4	180	2
c-c3-c3-c	9	1.4	0	3
o-c-os-c	2	5.4	180	2
os-c-c3-c3	6	0	180	2
os-c-c3-hc	6	0	180	2
os-c-c3-h1	6	0	180	2
o-c-c3-h1	1	0.8	0	-1
o-c-c3-h1	1	0	0	-2
o-c-c3-h1	1	0.08	180	3
c-c3-c3-h1	9	1.4	0	3
h1-c3-c3-hc	9	1.4	0	3
o-c-n-c3	4	10	180	2
o-c-n-hn	1	2.5	180	-2
o-c-n-hn	1	2	0	1
c3-c3-c3-h1	9	1.4	0	3
O-C-n-hn	1	2.5	180	-2
O-C-n-hn	1	2	0	1
O-C-n-c3	4	10	180	2
CT-C-n-hn	4	10	180	2
CT-C-n-c3	4	10	180	2

Impropers					
c3-n-c-o		10.5	180	2	
c-c3-n-hn		1.1	180	2	
c3-o-c-os		1.1	180	2	
**Nonbonding**			**Nonbonding**		
n	1.824	0.17	c	1.908	0.086
hn	0.6	0.0157	os	1.6837	0.17
c3	1.908	0.1094	o	1.6612	0.21
hc	1.487	0.0157	h1	1.387	0.0157

Chn-C3 group					
**Atom name**	**Atom type**	**Charge**	**Atom name**	**Atom type**	**Charge**
N	n	-0.5709	Hβ2	hc	0.050402
HN	hn	0.405221	Cγ	c3	-0.077127
Cα	c3	0.178	Hγ3	hc	0.039385
Hα1	h1	0.059255	Hγ1	hc	0.039385
Hα2	h1	0.059255	Hγ2	hc	0.039385
Cβ	c3	-0.101962	Hβ1	hc	0.050402
Parameters:					
**Mass**			**Bond**		
n	14.01	0.53	hn-n	403.2	1.013
hn	1.008	0.161	c3-n	328.7	1.462
c3	12.01	0.878	c3-h1	330.6	1.097
h1	1.008	0.135	c3-c3	300.9	1.538
hc	1.008	0.135	c3-hc	330.6	1.097
			c-n	427.6	1.379
**Angle**			**Angle**		
h1-c3-n	49.8	108.88	c3-c3-h1	46.4	109.56
c3-c3-n	65.9	111.61	hc-c3-hc	39.4	107.58
c3-n-hn	45.8	117.68	o-c-n	74.2	123.05
c3-c3-hc	46.3	109.8	c-n-hn	48.3	117.55
c3-c3-c3	62.9	111.51	c3-c-n	63.4	120.69
h1-c3-h1	39.2	108.46	c-n-c3	74.3	112.82
**Dihedrals**					
hc-c3-c3-n		9	1.4	0	3
c3-c3-c3-n		9	1.4	0	3
h1-c3-n-hn		6	0	0	2
c3-c3-n-hn		6	0	0	2
c3-c3-c3-hc		1	0.16	0	3
h1-c3-c3-hc		9	1.4	0	3
c3-c3-c3-h1		9	1.4	0	3
hc-c3-c3-hc		1	0.15	0	3

o-c-n-hn		1	2.5	180	-2
o-c-n-hn		1	2	0	1
o-c-n-c3		4	10	180	2
c3-c-n-hn		4	10	180	2
c3-c-n-c3		4	10	180	2
**Impropers**					
**Nonbonding**			**Nonbonding**		
n	1.824	0.17	h1	1.387	0.0157
hn	0.6	0.0157	hc	1.487	0.0157
c3	1.908	0.1094			

**<u>Chn-C6 group</u>**					
**Atom name**	**Atom type**	**Charge**	**Atom name**	**Atom type**	**Charge**
N	n	-0.5709	Hδ2	hc	0.04547
HN	hn	0.393772	Hδ1	hc	0.04547
Cα	c3	0.172719	Cε	c3	-0.068714
Hα1	h1	0.057497	Hε2	hc	0.044325
Hα2	h1	0.057497	Hε1	hc	0.044325
Cβ	c3	-0.105464	Cζ	c3	-0.079568
Hβ2	hc	0.050052	Hζ1	hc	0.037834
Cγ	c3	-0.067004	Hζ2	hc	0.037834
Hγ1	hc	0.04318	Hζ3	hc	0.037834
Hγ2	hc	0.04318	Hβ1	hc	0.050052
Cδ	c3	-0.067004			
<u>Parameters:</u>					
**Mass**			**Bond**		
n	14.01	0.53	hn-n	403.2	1.013
hn	1.008	0.161	c3-n	328.7	1.462
c3	12.01	0.878	c3-h1	330.6	1.097
h1	1.008	0.135	c3-c3	300.9	1.538
hc	1.008	0.135	c3-hc	330.6	1.097
			c-n	427.6	1.379
**Angle**			**Angle**		
h1-c3-n	49.8	108.88	c3-c3-h1	46.4	109.56
c3-c3-n	65.9	111.61	hc-c3-hc	39.4	107.58
c3-n-hn	45.8	117.68	o-c-n	74.2	123.05
c3-c3-hc	46.3	109.8	c-n-hn	48.3	117.55
c3-c3-c3	62.9	111.51	c3-c-n	63.4	120.69
h1-c3-h1	39.2	108.46	c-n-c3	74.3	112.82
**Dihedrals**					
hc-c3-c3-n		9	1.4	0	3

c3-c3-c3-n		9	1.4	0	3
h1-c3-n-hn		6	0	0	2
c3-c3-n-hn		6	0	0	2
c3-c3-c3-hc		1	0.16	0	3
c3-c3-c3-c3		1	0.18	0	-3
c3-c3-c3-c3		1	0.25	180	-2
c3-c3-c3-c3		1	0.2	180	1
h1-c3-c3-hc		9	1.4	0	3
c3-c3-c3-h1		9	1.4	0	3
hc-c3-c3-hc		1	0.15	0	3
o-c-n-hn		1	2.5	180	-2
o-c-n-hn		1	2	0	1
o-c-n-c3		4	10	180	2
c3-c-n-hn		4	10	180	2
c3-c-n-c3		4	10	180	2
**Impropers**					
**Nonbonding**			**Nonbonding**		
n	1.824	0.17	h1	1.387	0.0157
hn	0.6	0.0157	hc	1.487	0.0157
c3	1.908	0.1094			

References

(1) Tena-Solsona, M.; Alonso-De Castro, S.; Miravet, J. F.; Escuder, B. Co-Assembly of Tetrapeptides into Complex PH-Responsive Molecular Hydrogel Networks. *J Mater Chem B* **2014**, *2* (37), 6192–6197

(2) Garw, P.; Sabatinib, A.; Vaccab, A. *Investigation of Equilibria in Solution. Determination of Equilibrium Constants with the HYPERQUAD Suite of Programs*, *Talanta*, **1996**, 43, 1739–1753.

(3) The PyMOL Molecular Graphics System, Version 3.0 Schrödinger, LLC.

(4) Wang, J.; Wang, W.; Kollman, P. A.; Case, D. A. Automatic Atom Type and Bond Type Perception in Molecular Mechanical Calculations. *J Mol Graph Model,* **2006**, *25* (2), 247–260.

(5) Jakalian, A.; Jack, D. B.; Bayly, C. I. Fast, Efficient Generation of High-Quality Atomic Charges. AM1-BCC Model: II. Parameterization and Validation. *J Comput Chem* **2002**, *23* (16), 1623-1641.

(6) Wang, J.; Wolf, R. M.; Caldwell, J. W.; Kollman, P. A.; Case, D. A. Development and Testing of a General Amber Force Field. *J Comput Chem.,* **2004**, *25* (9), 1157–1174.

(7) Case. D. A.; Aktulga H.M.; Belfon K.; Ben-Shalom I.Y.; Berryman J.T.; Brozell S.R. et.al, *AmberTools 24*; San Francisco (CA), University of California, **2025**.

(8) Jorgensen, W. L.; Chandrasekhar, J.; Madura, J. D.; Impey, R. W.; Klein, M. L. Comparison of Simple Potential Functions for Simulating Liquid Water. *J Chem Phys,* **1983**, *79* (2), 926–935.

(9) Phillips, J. C.; Braun, R.; Wang, W.; Gumbart, J.; Tajkhorshid, E.; Villa, E.; Chipot, C.; Skeel, R. D.; Kalé, L.; Schulten, K. Scalable Molecular Dynamics with NAMD. *J Comput Chem.*, **2005**, 26, 1781–1802.

(10) Duan, Y.; Wu, C.; Chowdhury, S.; Lee, M. C.; Xiong, G.; Zhang, W.; Yang, R.; Cieplak, P.; Luo, R.; Lee, T.; Caldwell, J.; Wang, J.; Kollman, P. A Point-Charge Force Field for Molecular Mechanics Simulations of Proteins Based on Condensed-Phase Quantum Mechanical Calculations. *J Comput Chem.,* **2003**, *24* (16), 1999–2012.

(11) Grest, G. S.; Kremer', K. Molecular dynamics simulation for polymers in the presence of a heat bath, *Phys. Rev. A*, **1986**, 33, 3628-3631.

(12) Darden, T.; York, D.; Pedersen, L. Particle Mesh Ewald: An N·log(N) Method for Ewald Sums in Large Systems. *J Chem Phys* **1993**, *98* (12), 10089–10092.

(13) Roe, D. R.; Cheatham, T. E. PTRAJ and CPPTRAJ: Software for Processing and Analysis of Molecular Dynamics Trajectory Data. *J Chem Theory Comput* **2013**, *9* (7), 3084–3095.

(14) Aqvist, J.; Luzhkov, V. B.; Brandsdal, B. O. Ligand Binding Affinities from MD Simulations. *Acc Chem Res* **2002**, *35* (6), 358–365.

(15) Dannenberg, J. J., An Introduction to Hydrogen Bonding. *J. Am. Chem. Soc.* **1998**, 120, 22, 5604.
